# Multi-cohort validation of Ascore: an anoikis-based prognostic signature for predicting disease progression and immunotherapy response in bladder cancer

**DOI:** 10.1186/s12943-024-01945-9

**Published:** 2024-02-10

**Authors:** Tianlei Xie, Shan Peng, Shujun Liu, Minghao Zheng, Wenli Diao, Meng Ding, Yao Fu, Hongqian Guo, Wei Zhao, Junlong Zhuang

**Affiliations:** 1 Department of Urology, Nanjing Drum Tower Hospital, The Affiliated Hospital of Nanjing University Medical School, Nanjing, 210008 China; 2 Department of Urology, Nanjing Drum Tower Hospital Clinical College of Nanjing Medical University, Nanjing, China; 3 Department of Pathology, Affiliated Drum Tower Hospital, Medical School of Nanjing University, Nanjing, China; 4 Department of Urology, Jiangsu Province Hospital of Chinese Medicine, Affiliated Hospital of Nanjing University of Chinese Medicine, Nanjing, China; 5 Department of Clinical Biochemistry School of Laboratory Medicine/Sichuan Provincial Engineering Laboratory for Prevention and Control Technology of Veterinary Drug Residue in Animal-Origin Food, Chengdu Medical College, No. 783, Xindu Rd, Chengdu, 610500 China

**Keywords:** Bladder cancer, Prognostic signature, Immunotherapy, Anoikis, Tumor immune microenvironment

## Abstract

Bladder cancer ranks as the 10th most common cancer worldwide, with deteriorating prognosis as the disease advances. While immune checkpoint inhibitors (ICIs) have shown promise in clinical therapy in both operable and advanced bladder cancer, identifying patients who will respond is challenging. Anoikis, a specialized form of cell death that occurs when cells detach from the extracellular matrix, is closely linked to tumor progression. Here, we aimed to explore the anoikis-based biomarkers for bladder cancer prognosis and immunotherapeutic decisions. Through consensus clustering, we categorized patients from the TCGA-BLCA cohort into two clusters based on anoikis-related genes (ARGs). Significant differences in survival outcome, clinical features, tumor immune environment (TIME), and potential ICIs response were observed between clusters. We then formulated a four-gene signature, termed "Ascore", to encapsulate this gene expression pattern. The Ascore was found to be closely associated with survival outcome and served as an independent prognosticator in both the TCGA-BLCA cohort and the IMvigor210 cohort. It also demonstrated superior predictive capacity (AUC = 0.717) for bladder cancer immunotherapy response compared to biomarkers like TMB and PD-L1. Finally, we evaluated Ascore’s independent prognostic performance as a non-invasive biomarker in our clinical cohort (Gulou-Cohort1) using circulating tumor cells detection, achieving an AUC of 0.803. Another clinical cohort (Gulou-Cohort2) consisted of 40 patients undergoing neoadjuvant anti-PD-1 treatment was also examined. Immunohistochemistry of Ascore in these patients revealed its correlation with the pathological response to bladder cancer immunotherapy (*P* = 0.004). Impressively, Ascore (AUC = 0.913) surpassed PD-L1 (AUC = 0.662) in forecasting immunotherapy response and indicated better net benefit. In conclusion, our study introduces Ascore as a novel, robust prognostic biomarker for bladder cancer, offering a new tool for enhancing immunotherapy decisions and contributing to the tailored treatment approaches in this field.

**Supplementary Information:**

The online version contains supplementary material available at 10.1186/s12943-024-01945-9.

## Introduction

Bladder cancer (BLCA) is a globally prevalent and challenging disease, ranking as the 10th most common tumor and the second most common urological tumor [1, 2]. It is notorious for its high incidence and recurrence rate. BLCA can be categorized into non-muscle-invasive bladder cancer (NMIBC) and muscle-invasive bladder cancer (MIBC), with approximately 25% of patients developing muscle infiltration as the disease progresses. Cisplatin-based neoadjuvant chemotherapy (NAC) followed by radical cystectomy (RC) is the standard treatment for localized MIBC. However, even post-surgery, many patients experience tumor recurrence and metastasis. Those with metastatic BLCA typically face a grim prognosis, with an average overall survival of just 12–14 months, even after undergoing platinum-based chemotherapy [3].

Recent advances in immunotherapy, particularly immune checkpoint inhibitors (ICIs), have shown promise in BLCA treatment. The US Food and Drug Administration (FDA) approved ICI (pembrolizumab) as a first-line therapy for advanced BLCA patients who were ineligible for platinum-based chemotherapy [4]. ICI (nivolumab) has been used as an adjuvant treatment for high-risk MIBC patients post-RC [5]. Moreover, neoadjuvant ICIs, with/without NAC preceding surgery, have demonstrated positive pathologic responses in multiple studies [6]. However, a significant challenge remains: identifying patients who will respond favorably to ICIs. Given that current biomarkers like PD-L1 and tumor mutational burden (TMB) lack consistent correlation with ICI response [1], there's an urgent need for reliable predictive tools in BLCA clinical practice.

Anoikis, a specific form of apoptosis, refers to programmed cell death triggered when cells detach from the extracellular matrix (ECM) or surrounding cells [7]. The initial step in cancer metastasis involves detachment from the ECM, enabling cells to become anchorage-independent and enter the lymphatic or blood circulation [8]. To metastasize and invade, cancer cells must develop resistance to anoikis through various pathways, thereby evading cell death. The significance of anoikis in tumor progression has been underscored in various cancers, including breast, lung, and pancreatic cancers [9–11]. In addition, recent research across various cancer types has revealed that prognostic models based on anoikis-related genes hold significant relevance for clinical prognosis and the immune microenvironment [12–14]. Particularly, studies in skin cutaneous melanoma and glioblastoma have highlighted the potential variability of these models in predicting immunotherapy responses in patient cohorts [15, 16]. While there are existing researches that provide insights into the role of anoikis in bladder cancer [17, 18], a notable gap remains: the predictive role of anoikis in relation to immunotherapy response in bladder cancer has not been thoroughly investigated. Furthermore, there is a need for additional studies to validate these prognostic models in real-world clinical settings. This underscores the importance of further exploration into the role of anoikis in bladder cancer, particularly in the context of its potential impact on treatment outcomes and patient response to immunotherapy.

**Fig. 1:**
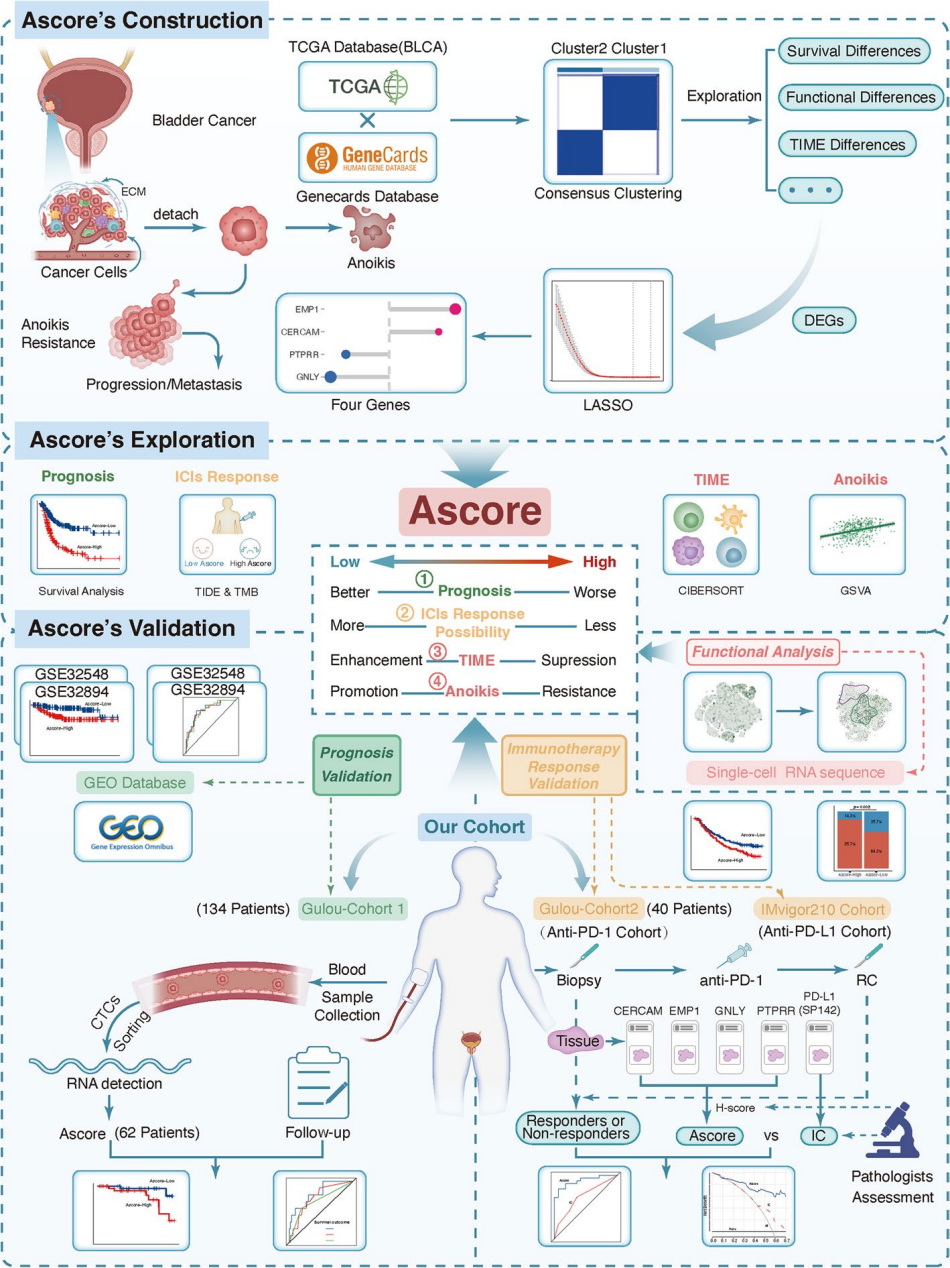
Flow chart of this study. The figure illustrates the experimental design and flow of this article. (Abbreviations: TCGA: The Cancer Genome Atlas; BLCA: Bladder Cancer; ECM: Extracellular Matrix; LASSO: Least Absolute Shrinkage and Selection Operator; DEGs: Differentially Expressed Genes; ICIs: Immune Checkpoint Inhibitors; TIME: Tumor Immune Microenvironment; TIDE: Tumor Immune Dysfunction and Exclusion; TMB: Tumor Mutation Burden; GSVA: Gene Set Variation Analysis; GEO: Gene Expression Omnibus; CTC: Circulating Tumor Cell; RC: Radical Cystectomy; FFPE: Formalin Fixation and Paraffin Embedding; IHC: Immunohistochemistry; IC: Immune Cell)

In this study, we employed RNA transcriptome analysis and single-cell RNA sequence analysis, along with various algorithms such as machine learning, immune infiltration, and enrichment analysis, to comprehensively explore the expression patterns of anoikis-related genes in bladder cancer. As a result, we introduced a prognostic signature, Ascore, to quantify these patterns and predict BLCA clinical outcomes and immunotherapy response. We further validated Ascore's potential as a pivotal BLCA biomarker using multiple cohorts, inclusive of our two retrospective ones. The experimental design is detailed in Fig. 1.

## Materials and methods

### Bulk RNA-seq data acquisition and preprocessing

Transcriptomic data and bladder cancer (BLCA) clinical profiles were sourced from The Cancer Genome Atlas (TCGA) database. Only samples with complete survival data were considered, with the best sample chosen for patients contributing multiple samples, ensuring patient-sample consistency. Our analysis encompassed 398 tumor samples and 18 normal adjacent samples. For validation, RNA-seq data and associated clinical details from bladder cancer cohorts (GSE32548, GSE32894) were extracted from the GEO (Gene Expression Omnibus) database. Duplicate gene symbols or multiple probes for a single gene were resolved by selecting the gene with the highest mean expression.

For dataset IMvigor210, RNA-seq and clinical information were accessed through R package IMvigor210CoreBiologies [19]. Our analysis focused on a subset of patients diagnosed with bladder cancer (*N* = 168), extracting from a larger pool of 298 patients, all of whom possessed complete treatment response information. Additionally, standardized microarray expression data for MIBC patients who received platinum-based NAC was obtained from the GSE169455 dataset [20].

A curated list of anoikis-related genes (ARGs) was derived from the GeneCards database, applying a relevance score threshold of greater than 1. Genes absent in the TCGA dataset were excluded, resulting in 332 ARGs for analysis.

### Consensus clustering and differential gene expression

Univariate Cox regression was employed to identify prognosis-associated ARGs. Based on these ARGs, consensus clustering was performed using the “ConsensusClusterPlus” R package [21]. Differential gene expression analysis was conducted using the “limma” package, with an absolute log fold change (|logFC|) > 1 and *P*-value < 0.05 as criteria [22].

### Prognostic ARG-based signature construction

To establish an ARG-based signature, we conducted a series of analyses involving univariate and multivariate Cox regression, as well as least absolute shrinkage and selection operator (LASSO) regression with tenfold cross-validation via the "glmnet" R package [23]. In LASSO regression, we selected “lambda.min” to prevent overfitting. A final set of 4 genes (CERCAM, EMP1, GNLY, PTPRR) was used to construct a prognostic formula, termed "Ascore".

$Ascore=\sum_{i=1}^{n}{Coe}_{i}*{Exp}_{i}$, where ${Coe}_{i}$ represents the coefficients of the genes and ${Exp}_{i}$ represents relative expression of genes in the cohort.

### Validation and performance assessment

Kaplan–Meier analysis was employed to compare the overall survival of high and low Ascore subgroups based on the median value. The accuracy of the Ascore in predicting survival at 1, 3, and 5 years was assessed using ROC curves via the “timeROC” R package [24].

### Prognostic nomogram construction

A prognostic nomogram incorporating the Ascore and other clinical features was constructed using the “rms” R package. The performance of the nomogram was evaluated through calibration curves and DCA.

### Single-cell RNA sequencing analysis

We analyzed normalized single-cell RNA sequencing (scRNA-seq) data from eight BLCA patients [25] with the “Seurat” R package [26]. To remove batch effects, data integration was performed using canonical correlation analysis (CCA) and mutual nearest neighbor (MNN) algorithms. The top 2,000 variable features were identified for each dataset, and integration was achieved using the “FindIntegrationAnchors” and “IntegrateData” functions. During this process, we excluded mitochondrial genes as they were not relevant to our study.

Next, we applied principal component analysis (PCA) to reduce the dimensionality of the integrated data, and the resulting first 30 principal components were set as input for dimensionality reduction using Uniform Manifold Approximation and Projection (UMAP) and t-Distributed Stochastic Neighbor Embedding (t-SNE) algorithms. Cell clusters were identified with the “FindClusters” function at a resolution of 0.4. Cell annotations were based on approaches described in the original literature.

For the sub-clustering of epithelial cells, we re-integrated the raw epithelial cell data and repeated the previously mentioned procedures. Differential gene expressions in subclusters were identified using the “FindMarkers” function, coupled with Wilcoxon rank-sum tests.

### Functional enrichment analysis

Functional enrichment analyses were performed via the “clusterProfiler” R package [27], focusing on GO (Gene Ontology) and KEGG (Kyoto Encyclopedia of Genes and Genomes) pathways. Additionally, Gene Set Enrichment Analysis (GSEA) and Gene Set Variation Analysis (GSVA) were performed to compare pathway activation between different groups [28]. All pathways were obtained from the MSigDB (The Molecular Signatures Database).

### Immune infiltration analysis

The ESTIMATE (Estimation of Stromal and Immune cells in Malignant Tumor tissues using Expression data) algorithm assessed variations in the immune score, stromal score, ESTIMATE score, and tumor purity among samples [29]. Additionally, the relative abundance of various cells was determined using the CIBERSORT and ssGSEA (single sample gene set enrichment analysis) methods via the “CIBERSORT” and “GSVA” R packages, respectively.

### Prediction of immunotherapy responsiveness

The Tumor Immune Dysfunction and Exclusion (TIDE) algorithm was used to predict responses to ICIs therapy in BLCA patients [30]. We also considered the expression levels of immune checkpoint genes (CD274, CTLA4, PDCD1, TIGIT, LAG3) and TMB as potential predictors for immunotherapy response. BLCA mutation data was obtained from the TCGA database, and TMB was computed using the 'maftools' R package [31].

### Study design and cohorts

We implemented a detailed retrospective analysis to investigate anoikis-related gene patterns in BLCA. Our study integrated 225 patients diagnosed with MIBC who underwent RC performed by a single experienced surgeon at the Drum Tower Hospital affiliated with Nanjing University (China) between 2017 and 2022. Informed consent was obtained from all patients, and the study protocol was approved by the Ethics Committee of Drum Tower Hospital to ensure ethical compliance. Rigorous follow-up procedures were implemented for these patients.

#### Gulou-Cohort1: Clinicopathological characteristics and prognosis validation cohort

This cohort involved 134 patients. The primary endpoint, overall survival (OS), was measured from RC to any-cause death. Prior to surgery, blood samples were collected using 2 CellSave Preservative tubes (CellSearch, 2 × 7.5 ml), with specific protocols for preservation.

Exclusion criteria: (1) Neoadjuvant therapy (NAT) preceding RC, and (2) Pathological diagnosis of non-urothelial carcinoma.

#### Gulou-Cohort2: Immunotherapy response validation cohort

Gulou-Cohort2 consists of 40 patients who received regular immunotherapy prior to RC. The primary observation endpoint was pathologic downstaging, classified into complete pathologic response (CR: pT0N0M0), pathologic downstage (PR: pTis, pTa, pT1 N0M0), and no downstaging (PD: pT2, pT3, pT4 with larger tumor volume or pN + , pM + ; SD: remaining status) [32], which was assessed by pathologists. The secondary endpoint, overall survival (OS), spanned from the start of immunotherapy to any-cause death.

Inclusion criteria: (1) MIBC diagnosis via bladder biopsy at Drum Tower Hospital, Nanjing University, and (2) At least two cycles of standardized Toripalimab (240 mg intravenously Q3W) anti-PD-1 immunotherapy.

Exclusion criteria: (1) Complete transurethral resection of the bladder tumor (TURBT) preceding immunotherapy, and (2) Pathological diagnosis of non-urothelial carcinoma.

#### CTC sorting and qRT-PCR detection

Blood collected from Gulou-Cohort1 participants underwent CELLSEARCH® Circulating Tumor Cell Kit manual. The CTC enrichment was performed by EpCAM-based immunomagnetic and automatic immunofluorescence staining. Next, experienced observers collected CTCs from sorting cells based on CD45-negative and with a diameter of at least 4 μm as previous report [33].

The total CTCs from a patient ≥ 5 were for further detection. We yield CTCs total RNA by commercial Dynabeads™ mRNA DIRECT™ Micro Purification Kit (Thermo). cDNA was synthesized according to the manufacturer’s instruction (QuantiNova Reverse Transcription Kit). CERCAM, EMP1, GNLY ,and PTPRR mRNA levels in CTCs was detected by PowerUp SYBR Green Master Mix (Thermo Fisher Scientific,) with primers: EMP1, GTGCTGGCTGTGCATTCTTG, and CCGTGGTGATACTGCGTTCC; CERCAM, GCACCGTTATGGGTACATGAA, and TGCTTCTAAGATCAGGTGGATGA; GNLY, CCTGTCTGACGATAGTCCAAAAA, and GACCTCCCCGTCCTACACA; PTPRR, TATACCAACACCACGGGAGAA, and AGTTCCATGACGCGGAATATC; β-Actin, CAAGATCAACCGGGAAAAGATGA, and TGGATGGCGACATACATGGC.

Gene expression levels were calculated as following equation:

$$2^{{\text{-}}\mathrm{\Delta Ct}}{\text{.}} [\mathrm{\Delta Ct}={\text{Ct}}({\text{target}})-{\text{Ct}}(\beta{\text{-Actin}})]$$

### Immunohistochemistry

For Gulou-Cohort2 participants, paraffin-embedded tissues were obtained prior to immunotherapy. Immunohistochemistry (IHC) staining was performed on 5 μm-thick sections. Sections were deparaffinized, rehydrated, and underwent antigen retrieval. A 5% bovine serum albumin (BSA) block preceded incubation with primary antibodies overnight at 4 °C, followed by secondary antibody incubation. Diaminobenzidine (DAB) visualized immunoreactivity. Amniotic coil containing a variety of commonly used control tissues were as positive and negative controls for antibody staining [34, 35].

To ensure unbiased evaluation, IHC results were independently assessed by two pathologists who were blinded to the clinical information, with any discrepancies resolved through discussion. The H-score method assessed staining intensity and extent, incorporating intensity (0 to 3 +) and the percentage of positively stained tumor cells (0% to 100%). For PD-L1, tumor-infiltrating immune cells (IC) scoring was implemented, categorizing IC0, IC1, IC2, or IC3 based on the percentage of PD-L1-positive ICs (< 1%, ≥ 1% but < 5%, ≥ 5% but < 10%, or ≥ 10%, respectively).

Primary antibodies used in IHC are listed as follows: CERCAM (Proteintech®, 16,411–1-AP, 1:400); EMP1 (CUSABIO®, CSB-PA007648LA01HU, 1:400); GNLY (CUSABIO®, CSB-PA009627LA01HU, 1:400); PTPRR (Proteintech®, 17,937–1-AP, 1:100); PD-L1 (SP142 using the UltraPATH platform).

### Statistical analysis

All data analysis was performed using R software (version 4.2.0). Comparisons between two independent groups were performed using a two-tailed Wilcoxon test if not specifically stated. For contingency table analysis, Fisher's exact test was utilized. Pearson correlation analyses were used to assess correlations between variables. Survival differences were evaluated using Kaplan–Meier (K-M) survival curves with log-rank tests. A *P* value < 0.05 was considered statistically significant.

## Results

### Mutation patterns and expression of ARGs in BLCA

In this study, we identified a total of 332 ARGs and analyzed their mutation prevalence across 398 BLCA patients. Notably, mutations in ARGs were detected in 385 of these individuals. Fig. S1A presents the top 10 mutated ARGs, signifying their pervasive role in BLCA pathogenesis. Particularly, TP53, PIK3CA, and RB1 emerged as the most frequently mutated ARGs. To delve into the implications of these mutations, we partitioned the BLCA patients into two distinct groups: the Wild and Mutant groups. GSVA was utilized to calculate the enrichment score of KEGG pathways in individual BLCA patients, and the ‘limma’ tool was employed to perform differential analysis. Results show that pathways upregulated in the Mutant group, including “Cell Cycle”, “DNA Replication”, and “Mismatch Repair” (Fig. S1B). Our data indicate potential defects in DNA repair, cell cycle regulation, and genomic stability associated with ARG mutations. Patients with mutations in ARGs may exhibit more aggressive tumor behavior.

To gain further insights into the involvement of ARGs in BLCA, we compared the 332 ARGs expression between normal and tumor bladder tissues using TCGA cohort. Our analysis revealed 112 genes upregulated in normal tissues and 86 in tumor tissues (Table S1). Univariate Cox regression yielded 54 prognosis-related ARGs (Fig. S1C), categorized as either 'risk' or 'protective' genes. The relationship between these 54 ARGs is visualized in Fig. S1D, showing the correspondent expression between 54 prognosis-related ARGs.

### Consensus clustering on ARGs in BLCA

**Fig. 2:**
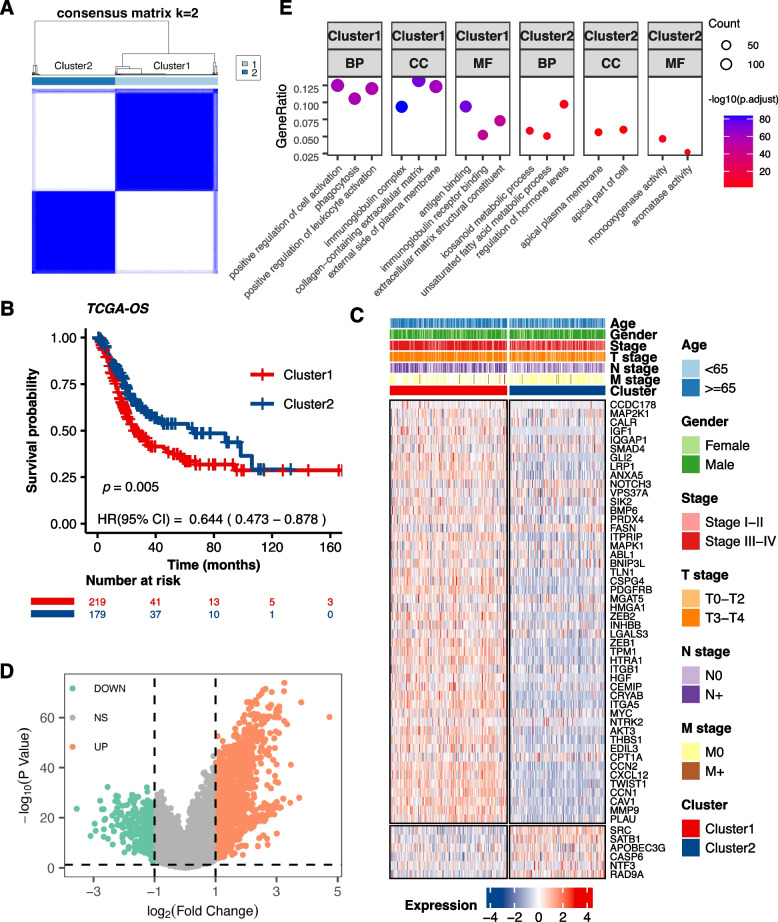
Consensus Clustering based on Prognostic Anoikis-Related Genes (ARGs) in BLCA. **A** Consensus matrix depicting the clustering results when k (cluster number) is set to 2. **B** Kaplan–Meier curves illustrating the overall survival differences between the two clusters (*P* = 0.005). **C** Heatmap displaying the expression levels of 54 prognostic ARGs, along with clinical characteristic annotations for each cluster. **D** Volcano plot of differentially expressed genes (DEGs) between clusters, with Cluster 2 as control (| logFC |> 1, *P* < 0.05). **E** Gene Ontology (GO) analysis highlighting the biological processes (BP), cellular components (CC), and molecular functions (MF) enriched between Cluster 1 and Cluster 2

To underscore the clinical relevance of ARGs, consensus clustering was conducted using the “ConsensusClusterPlus” R package, revealing two distinct patient clusters when k = 2 (Fig. 2A, Fig. S2A, B). Kaplan–Meier survival analysis confirmed significant survival differences between the clusters (*P* = 0.005; Fig. 2B). Clinical variables like age, pathological stage, and lymph node metastasis also exhibited pronounced variance between the two clusters (Table 1). Patients in Cluster1 were older, had higher stage grading, higher T grading, and were more likely to have lymph node metastasis. Importantly, the two clusters displayed divergent expression patterns of the 54 prognosis-related ARGs (Fig. 2C), with “risk” genes highly expressed in Cluster1, whereas “protective” genes in Cluster2.

**Table 1:** Clinical characteristics of two clusters

Characteristic	No (%)		*P* value
	Cluster.1 (*N* = 219)	Cluster.2 (*N* = 179)	
**Age**			
< *65*	72 (18.1%)	77 (19.3%)	
> = *65*	147 (36.9%)	102 (25.6%)	0.048 *
**Gender**			
*Female*	63 (15.8%)	39 (9.8%)	
*Male*	156 (39.2%)	140 (35.2%)	0.134
**Stage**			
*Stage I-II*	45 (11.3%)	83 (20.9%)	
*Stage III-IV*	174 (43.7%)	96 (24.1%)	< 0.001 ***
**T stage**			
*T0-T2*	47 (11.8%)	72 (18.1%)	
*T3-T4*	172 (43.2%)	107 (26.9%)	< 0.001 ***
**Lymph node metastasis**			
*No*	117 (32.9%)	114 (32%)	
*Yes*	88 (24.7%)	37 (10.4%)	< 0.001 ***
**Distant metastasis**			
*No*	77 (37.6%)	117 (57.1%)	
*Yes*	7 (3.4%)	4 (2%)	0.129

^†*^P < 0.05

^**^P < 0.01

^***^P < 0.001

Next, in order to gain insights into the molecular characteristics behind the distinction, 1520 differentially expressed genes (DEGs) between the clusters were identified (Fig. 2D). Functional analysis through GO and KEGG analysis (Fig. 2E, Fig. S2C) showed that Cluster1 was closely related to pro-invasive functions such as “positive regulation of cell activation” and “cytokine-cytokine receptor interaction”. On the other hand, Cluster2 may be associated with metabolic alterations. Additionally, GSVA revealed that Cluster1 was closely related to epithelial‐to‐mesenchymal transition (EMT) and nuclear factor‐κB (NF‐κB) signaling (Fig. S2D), both of which were linked to resistance to anoikis or apoptosis [36, 37]. These findings indicated significant differences in biological functions between the two clusters categorized by ARGs and demonstrated the rationality and implications of such categorization in bladder cancer.

### Exploring immune infiltration and responsiveness to immunotherapy between two clusters

**Fig. 3:**
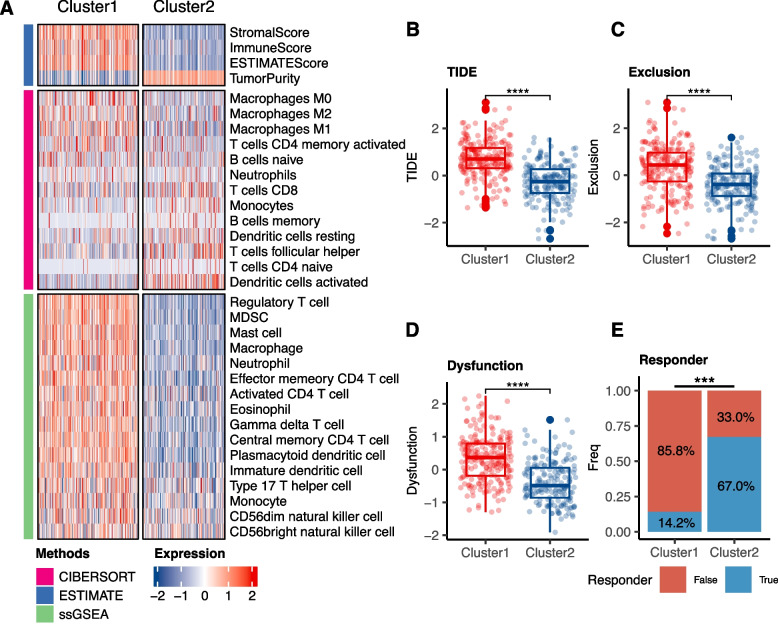
Immune Infiltration and Responsiveness to Immunotherapy across Clusters. **A** ESTIMATE scores and immune cell populations compared between Cluster 1 and Cluster 2. (B-E) TIDE analysis including TIDE score **B** Exclusion score **C **Dysfunction score **D** and potential immunotherapy responders **E** (*P < 0.05, **P < 0.01, ***P < 0.001, ****P < 0.0001)

The Tumor immune microenvironment (TIME) plays a critical role in cancer development and progression [38]. Variability in TIME among patients is closely linked to responsiveness to immunotherapy [39]. Despite limited research exploring the relationship between anoikis and TIME, understanding the TIME landscape in relation to different ARGs expression patterns in BLCA is crucial. Utilizing three algorithms: “ESTIMATE”, “CIBERSORT”, and “ssGSEA”, we depicted variations in TIME components between clusters (Fig. 3A). Cluster 1 displayed elevated Stromal, Immune, and ESTIMATE scores, yet had reduced Tumor Purity, presenting a more complex immune microenvironment characterized by increased tumor heterogeneity. Moreover, Cluster1 showed a higher abundance of immunosuppressive cells such as regulatory T cells and CD4 T cells, while Cluster2 exhibited higher expression of CD8 T cells, suggesting an active and potentially effective anti-tumor immune response. These findings indicate significant differences in the TIME between the clusters, with Cluster 1 being more complex.

TIDE is an algorithm designed to predict the response of immunotherapy, with a higher TIDE score meaning a reduced benefit from immunotherapy and an increased risk of immune escape. In our analysis, Cluster1 exhibited higher “TIDE scores”, “Exclusion scores”, and “Dysfunction scores” (Fig. 3B-D). Furthermore, Cluster2 had a higher proportion of patients predicted to respond to immunotherapy (*P* < 0.0001; Fig. 3E). These results support the notion that patients in Cluster2 may derive more benefit from immunotherapy compared to those in Cluster1.

### Establishment and validation of an anoikis-based signature

**Fig. 4:**
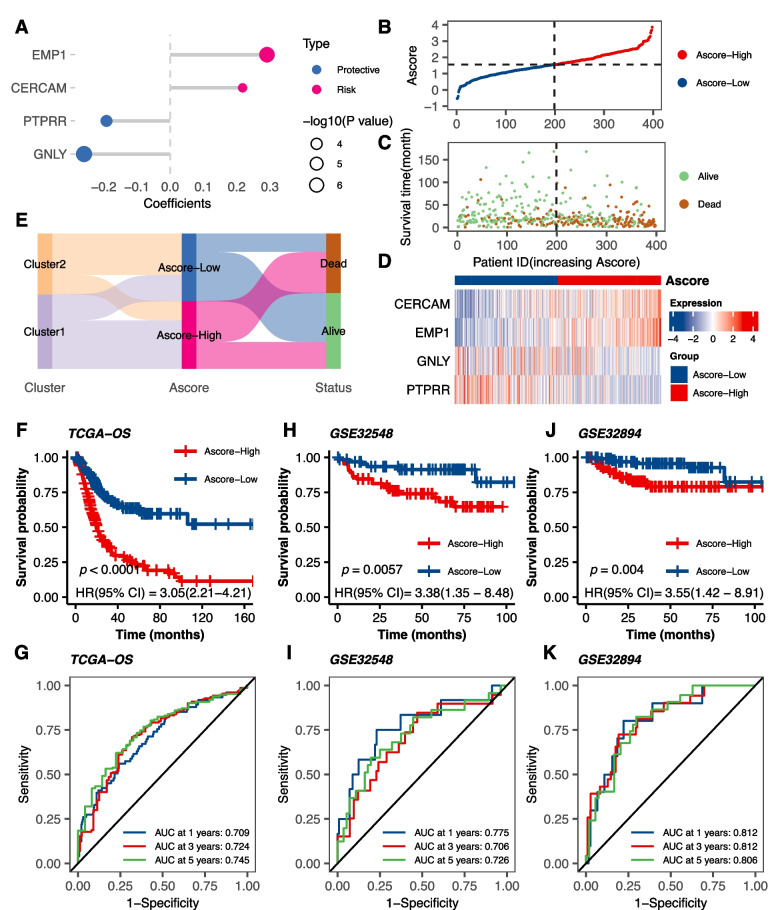
Establishment and Validation of the Ascore Prognostic Signature. **A** Multivariate Cox coefficients for four ARGs (CERCAM, EMP1, GNLY, PTPRR) in the prognostic signature. **B** Ascore distribution among BLCA patients, sorted from lowest to highest. **C** Survival status categorized by Ascore for each BLCA patient. **D** Heatmap displaying expression levels of four genes in different Ascore groups. **E** Sankey diagram correlating clusters, Ascore groups, and BLCA survival status. **F** Kaplan–Meier analysis comparing overall survival between high and low Ascore groups in BLCA (*P* < 0.0001). **G** Receiver Operating Characteristic (ROC) curves depicting Ascore signature’s predictive performance for 1, 3, and 5-year overall survival in BLCA, with the Area Under the Curve (AUC) values of 0.709, 0.724, and 0.745, respectively. (H–K) Kaplan–Meier analysis and time-dependent ROC curves in two external validation sets: GSE32548 and GSE32894

Recognizing the impact of ARGs on BLCA patient outcomes and immunotherapeutic responsiveness, a prognostic signature was pursued to understand bladder cancer's underlying complexities. Through univariate Cox regression analysis, 654 of the previously mentioned 1520 DEGs were identified as preliminary prognostic, which was narrowed down using the LASSO algorithm, leading to 16 genes (Fig. S3A, B). After subsequent multivariate Cox regression analysis (Fig. S3C), a prognostic signature called "Ascore" was constructed from four genes: CERCAM, EMP1, GNLY, and PTPRR (Fig. 4A). The formula is as follows: Ascore = (0.218 × CERCAM expression) + (0.291 × EMP1 expression) + (-0.259 × GNLY expression) + (-0.192 × PTPRR expression).

Next, we calculated Ascore for each individual patient in the BLCA cohort and sorted them from lowest to highest (Fig. 4B). Based on the median Ascore value, patients were divided into high and low Ascore groups. The survival status of the patients (Fig. 4C) and the expression levels of the 4 genes in the signature (Fig. 4D) were also displayed. Notably, patients with higher Ascore values correspond to worse survival outcomes, accompanied by higher expression levels of the "risk" genes, CERCAM and EMP1. To visually depict the relationship between clusters, Ascore subgroups, and survival status in BLCA, we utilized a Sankey diagram (Fig. 4E). The diagram revealed that patients with higher Ascore were more likely to be categorized in the Cluster1 and had a poorer prognosis. This observation was further supported by Kaplan–Meier analysis (Fig. 4F), with patients in the low Ascore group having significantly superior overall survival (OS, *P* < 0.0001). Similar trends were observed in other indicators such as Disease-Free Survival (DSS, *P* < 0.0001), Progression-Free Interval (PFI, *P* < 0.0001), and Disease-Free Interval (DFI, *P* = 0.0453) in BLCA (Fig. S3D-F).

To evaluate the predictive ability of our prognostic signature, we generated Receiver Operating Characteristic (ROC) curves for OS at 1, 3, and 5 years (Fig. 4G). The area under the curve (AUC) values were 0.709, 0.724, and 0.745, respectively, indicating the good predictive performance of our model, especially in long-term survival. Furthermore, we validated the accuracy of our signature in two independent external validation sets (GSE32548, GSE32894) and yielded satisfying results, with 5-years AUC values of 0.726 and 0.806, respectively (Fig. 4H-K).

To improve clinical utility, we also created a nomogram using Ascore and clinical parameters. Univariate and multivariate Cox regression analyses assessed the effects of clinical features and Ascore on BLCA survival. Both age and Ascore emerged as significant survival predictors (Fig. S4A). Using these predictors, we devised a nomogram estimating 1, 3, and 5-year survival probabilities for BLCA patients (Fig. S4B). Calibration curves attested to the nomogram's predictive accuracy (Fig. S4C-E). Decision curve analysis (DCA) affirmed its potential patient benefits (Fig. S4F). Overall, these suggest that the Ascore-based nomogram, rooted in anoikis concepts, has substantial clinical predictive value for bladder cancer.

### High Ascore indicates advanced disease and anoikis resistance

We further analyzed the relationship between Ascore and clinical features and found older patients as well as those with advanced disease often had higher Ascore values. (Fig. S5A). To determine the biological significance of Ascore, we screened out DEGs between Ascore groups (Fig. S5B) and performed KEGG analysis. The results showed that PI3K-AKT signaling pathway, which was reported to promote proliferation and inhibit anoikis in bladder cancer, was enriched in the high Ascore group (Fig. S5C). While the low Ascore group exhibited opposite with an abundance in the PPAR signaling pathway [40, 41]. Furthermore, anoikis-related gene sets from the GO database further validate our findings (Fig. S5D), with Ascore positively correlated with the “negative regulation of anoikis” while negatively correlated with the “positive regulation of anoikis”. Our findings illustrated that a high Ascore represents an anoikis-resistant status.

**Fig. 5:**
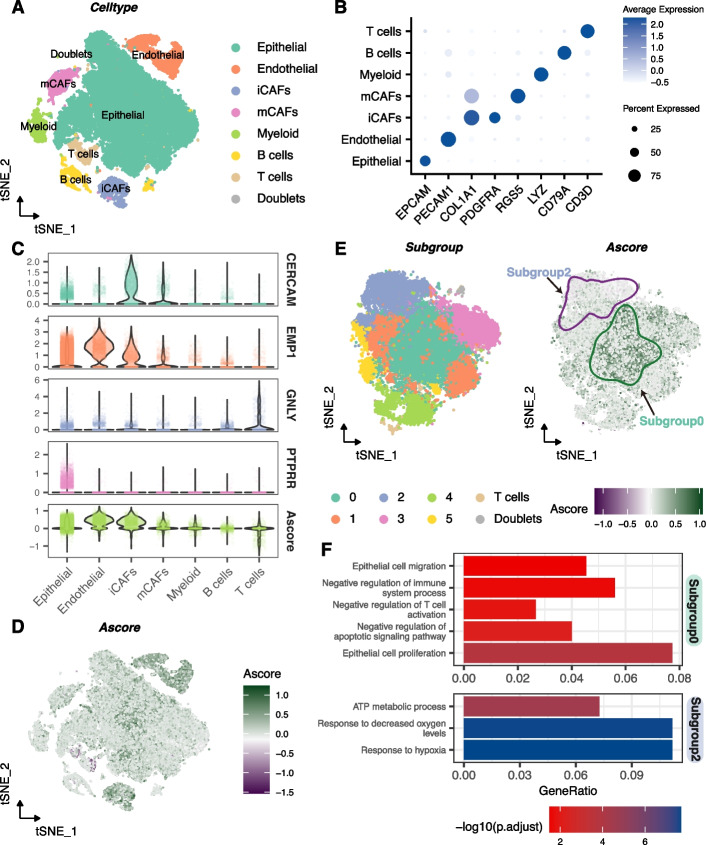
Single-Cell RNA Sequencing Analysis of Ascore Distribution and Biological Significance in Bladder Cancer. **A** t-SNE plot showing seven main cell types distribution in the integrated dataset, with doublets manually annotated. **B** Dot plot of marker genes' expression levels in each cell type. **C** Ascore and four genes (CERCAM, EMP1, GNLY, PTPRR) expression and distribution across cell types. **D** t-SNE plot showing Ascore expression levels and patterns in each cell type. **E** Left plot: Six main epithelial cell subgroups visualized by t-SNE dimensionality reduction. Right plot: Ascore distribution and expression in epithelial cells, highlighting Subgroup0 and Subgroup2. **F** GO analysis of biological function differences between Subgroup0 and Subgroup2

We further investigated using single-cell RNA sequencing data from HRA000212, which integrated 8 samples of bladder cancer for subsequent analysis (Fig. S6A). After manually eliminating doublet, a total of 41,387 cells were annotated into seven groups, as shown in the t-SNE and UMAP plot (Fig. 5A, B, Fig. S6B, C): Epithelial (EPCAM); Endothelial (PECAM1); iCAFs (COL1A1, PDGFRA); mCAFs (COL1A1, RGS5); Myeloid (LYZ); B cells (CD79A); T cells (CD3D).

We proceeded to explore the distribution of four genes comprising the Ascore in the dataset (Fig. 5C, Fig. S6D). Our findings revealed that CERCAM was predominantly expressed in inflammatory cancer-associated fibroblasts (iCAFs), a type of cancer-associated fibroblasts known for their strong pro-proliferation properties. On the other hand, EMP1 exhibited a wide distribution in bladder cancers but showed a particular concentration in endothelial cells. GNLY and PTPRR were primarily found in T cells and epithelial cells, respectively. Subsequently, we calculated the Ascore in each individual cell and observed endothelial cells and iCAFs exhibited higher levels. However, the distribution of Ascore varied significantly in epithelial cells (Fig. 5C, D, Fig. S6D), which sparked our curiosity.

We further sub-clustered epithelial cells into 6 groups (Fig. 5E), and observed high Ascore concentration in the Subgroup0, in contrast to the Subgroup2. Further biological function analysis elucidated the underlying differences. As shown in Fig. 5F, Subgroup0 epithelial cells exhibited a higher propensity for proliferation and migration, and displayed resistance to apoptosis. Moreover, these cells appeared to be involved in suppressing immune responses, particularly T cells activation. On the other hand, in Subgroup2, cells appeared to be in a hypoxic tumor microenvironment.

Collectively, our findings based on both RNA-seq and scRNA-seq proved that higher Ascore correlated with more aggressive and unfavorable phenotypes, and reflected anoikis resistance in bladder cancer.

### Immune landscape variations between different Ascore groups in TCGA-BLCA

Since Subgroup0 has shown an association with immunosuppression. We further explored the relationship between Ascore and TIME in the TCGA-BLCA cohort, finding immunosuppressive cells like CD4 + T cells dominated in the high Ascore group (Fig. S7A). On the contrary, CD8 + T cells and NK cells expression was abundant in the low Ascore group, indicating an immune active microenvironment.

TMB is a metric reflecting the number of mutations in cancer. Many researches have shown that high TMB predicts a more likely T-cell response and a greater likelihood of benefiting from treatment with ICIs [42]. In our study, high Ascore patients displayed lower TMB (*P* = 0.0034; Fig. S7B), indicating less potential benefit from immunotherapy. Additionally, the TIDE results substantiate this finding, with patients in the high Ascore group presenting higher TIDE scores (*P* < 0.0001; Fig. S7C) and a lower proportion of response (*P* < 0.0001, Fig. S7D).

### Evaluating Ascore as a prognostic tool and predictor of immunotherapy response

**Fig. 6:**
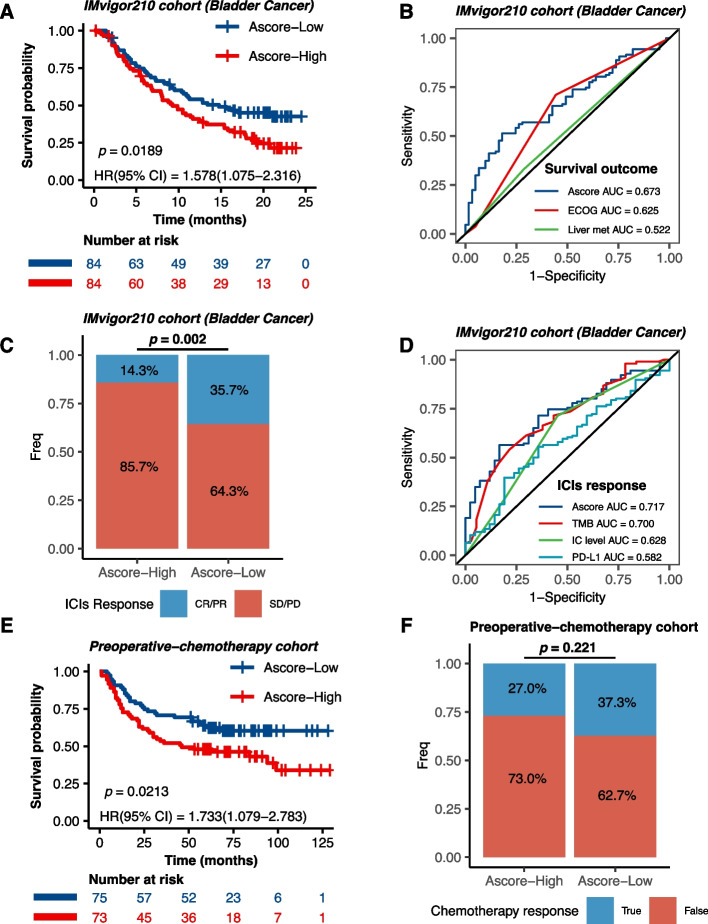
Ascore's Prognostic and Predictive Role in Immunotherapy and Chemotherapy Cohorts. **A** Survival outcomes within post-immunotherapy bladder cancer patients relative to different Ascore groups. **B** ROC analysis of Ascore's predictive performance for survival against other prognostic factors like ECOG and liver metastasis in bladder cancer. **C** Response rates to immunotherapy in bladder cancer patients based on Ascore groups. **D** ROC analysis illustrating Ascore’s predictive accuracy for immunotherapy response in bladder cancer. **E** Ascore’s prognostic significance in chemotherapy-treated MIBC patients. **F** Assessment of Ascore in predicting pathological response to chemotherapy. (ECOG: Eastern Cooperative Oncology Group; ICIs: Immune Checkpoint Inhibitors; PD: progressive disease; SD: stable disease; PR: partial response; CR: complete response; TMB: Tumor Mutational Burden; IC: Immune Cell)

To comprehensively assess the relationship between Ascore and immunotherapy, we turned to the IMvigor210 cohort, a well-known large phase II clinical trial investigating treatment response to anti-PD-L1 (atezolizumab) immunotherapy in patients with advanced urothelial cancer. Here, we noticed that a higher Ascore was associated with adverse survival outcomes post-immunotherapy (*P* = 0.0189; Fig. 6A). We incorporated additional risk factors, such as gender, Eastern Cooperative Oncology Group (ECOG) performance status, liver metastases presence, and smoking history, into our prognostic evaluation. Both univariate and multivariate analyses positioned Ascore as an independent prognostic indicator (Fig. S8A) with robust predictive performance for survival (AUC = 0.673; Fig. 6B), outperforming other known prognostic factors. Intriguingly, this trend remained consistent when we broadened our analysis to include all patients with urothelial cancer receiving immunotherapy within the cohort (Fig. S8B-D).

We also noticed that divergent Ascore expressions were associated with varying responses to immunotherapy, with a significant proportion of low Ascore bladder cancer patients responding favorably (*P* = 0.002; Fig. 6C). We then evaluated the ability of Ascore to predict immunotherapy response in bladder cancer, and the ROC analysis (Fig. 6D) illustrated Ascore’s superior predictive capacity (AUC = 0.717) compared to TMB (AUC = 0.700), PD-L1 expression levels on immune cells (AUC = 0.628), and PD-L1 expression in tumor tissues (AUC = 0.582). Similar trends were found in the entire urothelial cancer cohort (Fig. S8E, F). These findings suggested Ascore’s utility as both a prognostic factor and a potential predictor of immunotherapy response.

Furthermore, to discern whether Ascore’s predictive ability was exclusive to immunotherapy, we included a group of 149 MIBC patients who were administered preoperative platinum-based neoadjuvant chemotherapy. We found that Ascore, while prognostically significant (*P* = 0.0213; Fig. 6E), did not anticipate the pathological response to chemotherapy (*P* = 0.221; Fig. 6F). This finding provides unique insights into Ascore’s specificity, implying a more pronounced predictive capability in the realm of immunotherapy response.

### Validation of Ascore as a non-invasive prognostic biomarker in circulating tumor cells

**Fig. 7:**
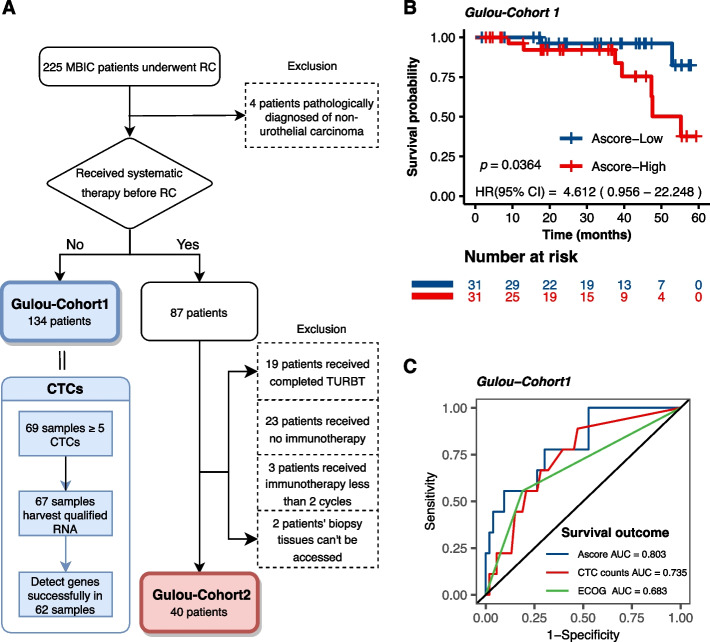
Study Design and Ascore Prognostic Validity Evaluation in Gulou-Cohort1. **A** Retrospective study stratification flowchart showing Gulou-Cohort1 (134 patients) and Gulou-Cohort2 (40 patients) compositions. Circulating tumor cells were successfully isolated and quantified from 62 Gulou-Cohort1 patients' blood samples. **B** Kaplan–Meier curves comparing prognostic outcomes between the high and low Ascore groups in Gulou-Cohort1. **C** ROC analysis showing Ascore’s predictive value versus CTC counts and ECOG score in Gulou-Cohort1. (CTC: Circulating tumor cell; ECOG: Eastern Cooperative Oncology Group)

To evaluate the real-world utility of Ascore in prognosticating survival and immunotherapy response in patients with bladder cancer, we conducted a retrospective investigation at Drum Tower Hospital, affiliated with Nanjing University in China. Patients who were diagnosed with bladder cancer and underwent RC were included in our analysis. These patients were stratified into two distinct cohorts, termed Gulou-Cohort1 and Gulou-Cohort2, as depicted in Fig. 7A.

Circulating tumor cells (CTCs) are neoplastic cells that have disseminated from the primary tumor site into the circulatory system and are implicated as a prognostic indicator in cancer progression and metastasis [43]. Within Gulou-Cohort1, we enrolled 134 patients who had not received any form of systemic therapy prior to RC. Blood samples were prospectively obtained, leading to the successful isolation and characterization of CTCs in 62 cases (Fig. 7A). Relative RNA expression of four specific genes (CERCAM, EMP1, GNLY, PTPRR) was quantified to compute the Ascore for each patient, subsequently categorizing them into Ascore-High and Ascore-Low groups (Table S2). Importantly, the Ascore-High group was predominantly comprised of older individuals, exhibited more advanced pathological stages, and had a greater likelihood of lymph node metastases (Table 2), but there was no significant association with ECOG score. These observations are congruent with our prior research. Moreover, higher Ascore values in CTCs were associated with worse prognostic outcomes compared to the Ascore-Low group (*P* = 0.0364; Fig. 7B).

**Table 2:** Clinical characteristics of high and low Ascore groups in Gulou-Cohort1

Characteristic	No (%)		*P* value
	Ascore-High (*N* = 31)	Ascore-Low (*N* = 31)	
**Age**			
< *65*	6 (9.7%)	14 (22.6%)	
> = *65*	26 (41.9%)	16 (25.8%)	0.029 *
**Gender**			
*Female*	11 (17.7%)	14 (22.6%)	
*Male*	21 (33.9%)	16 (25.8%)	0.438
**Stage**			
*Stage II*	12 (19.4%)	22 (35.5%)	
*Stage III*	20 (32.3%)	8 (12.9%)	0.006 **
**T stage**			
*T2*	14 (22.6%)	23 (37.1%)	
*T3*	18 (29%)	7 (11.3%)	0.011 *
**Lymph node metastasis**			
*No*	23 (37.1%)	28 (45.2%)	
*Yes*	9 (14.5%)	2 (3.2%)	0.044 *
**ECOG score**			
*0*	22 (35.5%)	25 (40.3%)	
*1*	9 (14.5%)	6 (9.7%)	0.554

^†^*ECOG* Eastern Cooperative Oncology Group

^*^P < 0.05

^**^P < 0.01

^***^P < 0.001

To further substantiate the prognostic significance of Ascore in CTCs, we included clinical parameters from Table 2 in a univariate Cox regression analysis and also integrated CTC count for a comparative assessment (Fig. S9). The CTC count, previously established as a significant prognostic marker in various cancers such as prostate cancer and small-cell lung cancer [44, 45], predicts disease outcomes effectively. The univariate Cox regression analysis identified lymph node metastasis, ECOG score, CTC count, and Ascore as risk factors for Gulou-Cohort1 (*P* < 0.05). Subsequent multivariate regression analysis revealed that both Ascore and ECOG score were independent prognostic factors (Fig. S9). We then evaluated the predictive capabilities of Ascore, CTC count, and ECOG score on the prognosis of Gulou-Cohort1 patients. The ROC curve demonstrated that Ascore, with an AUC of 0.803, outperformed the CTC technique (AUC = 0.735) and ECOG score (AUC = 0.683) in prognostic prediction (Fig. 7C). This validation within our cohort underscores Ascore's potential as a non-invasive prognostic marker and confirms its clinical applicability.

### Ascore predicts anti-PD-1 immunotherapy response in Gulou-Cohort2

**Fig. 8:**
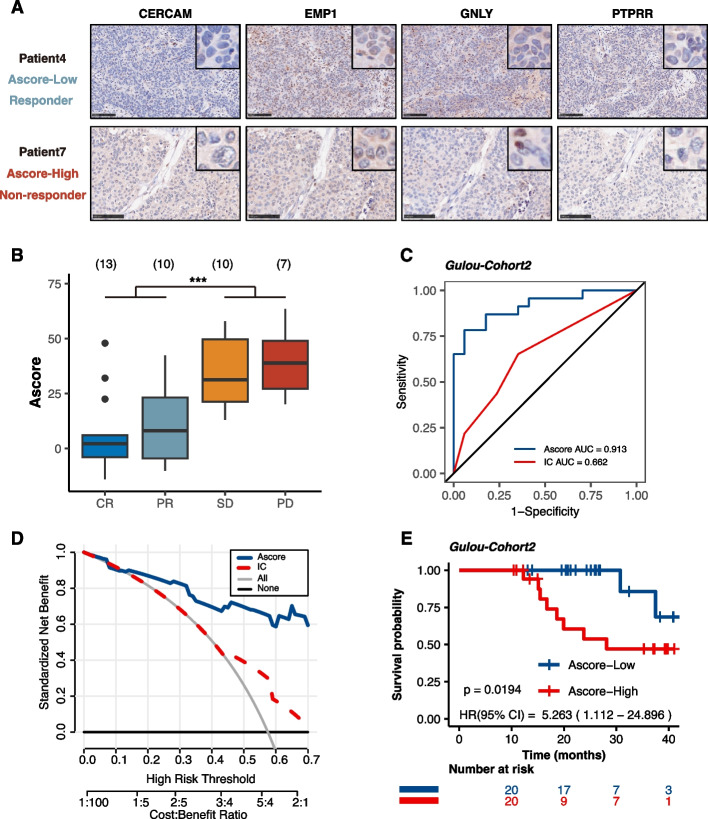
Ascore Predictive Capability for Anti-PD-1 Immunotherapy Response in Gulou-Cohort2. **A** Representative immunohistochemistry (IHC) images illustrating the expression of four key genes (CERCAM, EMP1, GNLY, PTPRR) in two patients from Gulou-Cohort2 (Scale bars = 100 μm). Patient 4, who responded to anti-PD-1 therapy, had a low Ascore, in contrast to non-responder Patient 7, who had a high Ascore. **B** Distribution of Ascores among different response groups (CR: complete response; PR: partial response; SD: stable disease; PD: progressive disease; ***P < 0.001). **C** ROC curves comparing the predictive accuracy of Ascore (AUC = 0.913) versus PD-L1 expression in tumor-infiltrating immune cells (ICs) (AUC = 0.662). **D** Decision curve analysis (DCA) indicating the net benefit of using Ascore compared to evaluating ICs' PD-L1 expression. **E** Kaplan–Meier curves c showing a correlation between higher Ascore values in tissue samples and reduced survival rates (*P* = 0.0194)

Our preceding study proposed that Ascore could predict the responsiveness of anti-PD-L1 immunotherapy in bladder cancer patients. To extend this finding to anti-PD-1 immunotherapy, we analyzed 40 patients who underwent standard anti-PD-1 treatment prior to RC. Pre-treatment tumor tissue was evaluated for the expression of the four aforementioned genes through immunohistochemistry (IHC). Each sample received an H-score, with the scoring criteria for the expression of these genes in tumor cells detailed in Fig. S10A. Individual Ascore values were derived using a previously established formula, as exhibited in Table S3. Figure 8A showed representative IHC images from the Ascore-Low (Patient4) and Ascore-High (Patient7) groups, illustrating a responder and a non-responder, respectively. We found significant differences in pathological response rates between the Ascore-High and Ascore-Low groups (*P* = 0.004; Table 3). Specifically, lower Ascore values in pre-treatment tumor samples were linked to a more favorable immunotherapy response (45% vs. 12.5%, *P* < 0.001). In addition, a higher rate of complete response (CR) was observed in the Ascore-Low group compared to the Ascore-High group (25% vs. 7.5%, *P* = 0.041). Consistently, patients who responded to immunotherapy (CR/PR) had noteworthy lower Ascores (*P* < 0.0001). Our results revealed a strong relation between Ascore and anti-PD-1 immunotherapy response.

**Table 3:** Pathologic response of high and low Ascore groups in Gulou-Cohort2

Characteristic	No (%)		*P* value
	Ascore-High (*N* = 20)	Ascore-Low (*N* = 20)	
**Pathological response**			
*CR*	3 (7.5%)	10 (25%)	
*PR*	9 (22.5%)	1 (2.5%)	
*SD*	3 (7.5%)	7 (17.5%)	
*PD*	5 (12.5%)	2 (5%)	0.004 **
**Binary response**			
*Responder (SD* + *PD)*	5 (12.5%)	18 (45%)	
*Non-responder (CR* + *PR)*	15 (37.5%)	2 (5%)	< 0.001 ***
**CR response**			
*CR*	3 (7.5%)	10 (25%)	
*Non-CR*	17 (42.5%)	10 (25%)	0.041 *

^†^*CR* complete response, *PR* partial response, *SD* stable disease, *PD* progression disease

^†,*^P < 0.05

^**^P < 0.01

^***^P < 0.001

To further refine the predictive accuracy of the Ascore signature, we expanded our analysis to include PD-L1 expression in tumor-infiltrating immune cells (ICs) as shown in Fig. S10B. These ICs—comprising macrophages, dendritic cells, and lymphocytes—are considered instrumental in shaping immunotherapy response in bladder cancer. Fig. S10C presents representative IHC images for Patient4 and Patient7, with their PD-L1 expression on ICs being IC2 and IC3, respectively. This challenges the common belief that higher PD-L1 levels correlate with a successful immunotherapy response. ROC analysis further indicated the predictive capability of Ascore (AUC = 0.913) was superior to that of ICs (AUC = 0.662) in forecasting immunotherapy pathologic response (Fig. 8C). DCA curves suggested that the net benefit of the Ascore was obviously higher than PD-L1 expression in ICs (Fig. 8D).

In alignment with data from Gulou-Cohort1, elevated Ascore values in tissue samples correlated with poorer survival rates, reinforcing the general applicability of Ascore as a predictive biomarker for bladder cancer progression (*P* = 0.0194, Fig. 8E).

## Discussion

Recent advancements in the diagnosis and treatment of bladder cancer have been noteworthy, yet survival rates for muscle-invasive bladder cancer remain suboptimal due to persistent challenges such as metastasis and recurrence. Anoikis, a specialized form of apoptosis, plays a pivotal role in cancer progression and metastasis. Studies have increasingly underscored the importance of anoikis in bladder cancer. For instance, a prognostic model comprising 7 long non-coding RNAs (lncRNAs) has illuminated the prognostic significance of anoikis in bladder cancer [18]. Additionally, another prognostic model involving 9 genes has elaborated on the association between anoikis and the immune microenvironment in bladder cancer [17]. Despite these important findings, the research field still faces significant limitations, such as the lack of validation in real-world clinical cohorts and the complexity of multi-gene models hindering their clinical application. Notably, the existing model has shown no significant correlation in the IMvigor210 cohort when it comes to immunotherapy, as illustrated in Fig. S11. Furthermore, the connection between anoikis and immunotherapy in cancer, although explored in various studies (as detailed in Table S4), remains to be firmly established in clinical settings. This highlights the need for further investigation to elucidate the role of anoikis in bladder cancer, especially in terms of its potential influence on treatment strategies and immunotherapeutic response.

In this study, we first identified 54 prognosis-related ARGs, segregating bladder cancer patients into two distinct clusters in TCGA cohort. These clusters diverged significantly in clinical characteristics, survival outcomes, biological functionalities, and immune profiles. Notably, Cluster1 showed relevance to epithelial-mesenchymal transition (EMT), a phenomenon wherein epithelial cells forfeit their polarity, diminishing intercellular adhesion, and adopt a mesenchymal phenotype [36]. This transformation predisposes tumor cells to increased migration, invasion, and heightened resistance to anoikis [8]. Furthermore, the presence of extracellular matrix components, particularly collagen, in Cluster1 is noteworthy due to its association with anoikis resistance in various cancer types [46, 47]. Our findings emphasize the integral connection between anoikis and the functional disparities observed in these clusters.

The pivotal role of TIME in cancer development and progression is well acknowledged, with a growing consensus that understanding its dynamics could pave the way for more targeted and effective immunotherapies [39]. Our exploration of the TIME in BLCA through the lens of different ARGs expression patterns added a new dimension to this ongoing discourse. Cluster 1 exhibited a more complex immune microenvironment characterized by a higher abundance of immunosuppressive cells including CD4 T cells and regulatory T cells, suggesting a landscape ripe for immune evasion and potentially reduced responsiveness to immunotherapies [48]. The higher TIDE, exclusion, and dysfunction scores further corroborate this. Contrastingly, Cluster 2 showcased a potentially more favorable landscape for immunotherapy, characterized by a higher expression of CD8 T cells, which are generally associated with effective anti-tumor immune responses. Our analysis sheds light on the heterogeneous landscape of TIME in BLCA, highlighting distinct clusters with differing potentials for immunotherapy responsiveness.

Next, we developed a prognostic signature, named "Ascore", to quantify our classification based on ARGs expression pattern. Ascore comprises four genes (CERCAM, EMP1, GNLY, and PTPRR), which have been previously reported to be strongly associated with cancer. CERCAM, an adhesion molecule, is associated with poor prognosis in bladder cancer and enhances tumor cell proliferation and invasion [49]. Remarkably, our research revealed the specific accumulation of CERCAM in iCAFs through single-cell RNA sequencing analysis. EMP1, a member of epithelial membrane proteins (EMPs) family, plays an important role in cancer invasion and metastasis [50]. High levels of EMP1 expression in bladder cancer contribute to lower overall survival rates and are strongly correlated with immune cell infiltration [51]. However, conflicting findings suggested EMP1 functions as a tumor suppressor in bladder cancer [52], highlighting the need for further research. GNLY, also known as granulysin, is predominantly found in immune cells such as T cells and NK cells. Recent studies have revealed its involvement in tumor immunity, leading to a more favorable prognosis [53]. The protein encoded by PTPRR belongs to the protein tyrosine phosphatase (PTP) family, which exhibits tumor suppressive properties [54]. However, there is limited research on the relevance of PTPRR in bladder cancer, which warrants further investigation in the future.

The resulting Ascore signature demonstrated evident efficacy across the BLCA cohort and two independent external GEO validation sets. Patients with a higher Ascore resembled characteristics in Cluster1 and were prone to have a bad prognosis. We subsequently validated the prognostic role of Ascore in an immunotherapy cohort and a neoadjuvant chemotherapy cohort, respectively, and obtained consistent findings. Through the validation of multiple cohorts above, we confirmed that Ascore can be used as a general bladder cancer prognostic marker.

To further explore the clinical application of Ascore, we conducted analyses on CTCs in our patient cohort. CTCs offer a unique window into tumor progression and metastasis, making them invaluable for cancer research [55]. While few current markers are clinically validated to predict bladder cancer progression using CTCs, our results indicated a compelling association between lower Ascore levels in CTCs and longer patient survival. Even after incorporating multiple clinical parameters such as CTC counts and ECOG, Ascore in CTCs remains an independent prognostic factor. This suggests that Ascore could serve as a potential, noninvasive prognostic biomarker in the field.

To elucidate the biological significance of Ascore, we categorized patients in the TCGA cohort into high and low Ascore groups. Our functional analysis revealed notable differences between the two. The high Ascore group showed enrichment in focal adhesion and the PI3K-AKT signaling pathway, both of which are linked to anoikis resistance and cell proliferation [37, 40, 56]. Conversely, the low Ascore group was abundant in PPAR signaling, which plays a contrasting role by inhibiting PI3K-AKT signaling and promoting cell apoptosis [41, 57]. Additionally, single-cell RNA sequencing analysis further substantiated these findings, revealing that epithelial cells with elevated Ascore expression were more likely to proliferate, resist anoikis, and suppress T-cell immune responses. Collectively, higher Ascore reflected a more aggressive tumor phenotype and a more immunosuppressive TIME where malignant cells are apt to evade immune surveillance.

Immunotherapy, particularly ICIs, has been commonly used as an adjuvant treatment for patients with bladder cancer who are unfit for platinum-based chemotherapy [48]. Additionally, ICIs applied as neoadjuvant therapy for MIBC patients before they receive RC have shown promising results [58]. However, some patients gained pronounced treatment responses to immunotherapeutic intervention while a large proportion experienced little or no benefit. Understanding the factors influencing immunotherapy efficacy is crucial. The treatment response, typically referring as a short-term reduction in tumor size or pathologic downgrading, and side effects, are key determinants [59]. Accurately predicting which patients will gain prompt response to immunotherapy is essential to tailor treatment strategies and minimize exposure to ineffective therapies. Our analysis of the IMvigor210 cohort, a single-arm, phase II trial focused on immunotherapy, revealed Ascore's predictive relationship with treatment response in both bladder and urothelial cancer patients. Ascore's predictive accuracy for immunotherapy response was found to be superior to TMB, Immune Cell (IC) levels, and PD-L1 expression. Due to the absence of two-arm clinical cohort data for a direct comparison, we utilized a single-arm chemotherapy cohort for indirect validation. This approach, although limited, suggested that Ascore's predictive capacity is more relevant to immunotherapy.

To bridge the gap between bioinformatics and clinical application, we extended our analysis to a retrospective cohort of patients undergoing pre-operative anti-PD-1 immunotherapy. The Ascore for each patient was assessed via IHC and independently scored by two experienced pathologists, ensuring the reliability of our evaluation. To further enhance the clinical applicability of our model, we utilized an amniotic coil encompassing a range of control tissues, serving as both positive and negative controls for the four antibodies employed in our study (Fig. S12). This approach enhanced the robustness of our methodology. Consequently, the findings were consistent, showing a lower Ascore associated with a better response to anti-PD-1 immunotherapy. Notably, the predictive accuracy of Ascore exceeded that of established markers like PD-L1, underscoring its potential in guiding immunotherapy choices for bladder cancer patients. This revelation positions Ascore as a promising tool in the personalized treatment landscape, particularly in the selection of suitable immunotherapy strategies.

Building on these promising results, our study's Ascore model not only complements established clinical scores like the Glasgow Prognostic Score and Bellmunt Score but also offers distinct advantages [60, 61]. While these clinical indicators provide broad prognostic insights based on systemic responses, Ascore focuses on a concise set of anoikis-related genes, enhancing its specificity and practicality for clinical use. Its robust validation across various datasets, including the IMvigor210 cohort and our own, underscores its effectiveness in predicting immunotherapy responses. This positions Ascore as a pivotal tool in personalized medicine for bladder cancer, offering rapid and precise prognostic information to aid informed clinical decision-making.

However, it is important to acknowledge the limitations of our approach. The most significant constraint stems from our reliance on data from a single-arm chemotherapy cohort, which, despite providing initial insights, does not offer the comprehensive validation attainable from a two-arm immunotherapy cohort. We recognize this gap and plan to address it in future studies by incorporating a wider range of data sources, aiming to reinforce the model’s validity and reliability. Additionally, the clinical application of the Ascore model, particularly involving techniques like circulating tumor cells and immunohistochemistry, poses practical challenges. The current process, albeit effective, can be labor-intensive, and the variability in judgment criteria raises concerns about its routine use in clinical settings. We are actively working towards simplifying and standardizing this process, collaborating with clinical experts to refine the evaluation criteria, thus making the Ascore model more accessible and applicable in clinical practice.

## Conclusion

In conclusion, our study provides valuable insights into the expression patterns and roles of anoikis-related genes in bladder cancer. The Ascore signature stands as a potent predictor of tumor progression and a potential guide in personalized clinical decision-making, particularly regarding immunotherapeutic strategies for bladder cancer. Nonetheless, broader validation of Ascore in additional bladder cancer cohorts is essential.

### Supplementary Information

**Additional file 1:** **Figure S1.** Mutation Patterns and Expression of Anoikis-related Genes (ARGs) in BLCA. (A) Top 10 mutated ARGs in the BLCA samples with mutations. (B) GSVA result showing differences in KEGG pathways between the Wild and Mutant groups. Pathways in blue/green indicate upregulation in the Mutant/Wild groups, respectively. (C) Forest plot of 54 prognostic ARGs. (D) Correlation plot illustrating interactions among the 54 prognosis-related ARGs. (*P < 0.05, **P < 0.01, ***P < 0.001).

** Additional file 2:** **Figure S2.** Consensus Clustering and inter-Cluster Differences. (A) Cumulative distribution function (CDF) plots illustrating consensus distribution with varying k values. (B) Delta area plot for relative change in the area under CDF curve. (C) KEGG analysis comparison between the two clusters. (D) GSEA results comparison, using Cluster 2 as the control group.

** Additional file 3:** **Figure S3.** Construction of an ARGs-based Prognostic Signature. (A-B) LASSO regression with 10-fold cross-validation identified 16 prognostic ARGs following univariate Cox regression analysis. (C) Multivariate Cox regression analysis of the 16 genes shortlisted by LASSO regression. (D-F) Kaplan-Meier analysis comparing Disease-Free Survival (DSS) (D), Progression-Free Interval (PFI) (E), and Disease-Free Interval (DFI) (F) between high and low Ascore groups in BLCA. (*P < 0.05, **P < 0.01).

** Additional file 4:** **Figure S4.** BLCA Survival Prediction Nomogram Based on Ascore. (A) Univariate Cox regression analysis of clinical characteristics and Ascore. Factors with *P* < 0.05 were included in subsequent multivariate Cox regression analysis. (B) Nomogram incorporating age and Ascore, utilized for 1, 3, and 5-year survival predictions. (C-E) Calibration curves at 1, 3, and 5 years, respectively, demonstrating nomogram's predictive accuracy. (F) Decision curve analysis (DCA) evaluating the clinical utility of the nomogram. (**P< 0.01, ***P < 0.001).

** Additional file 5:** **Figure S5.** High Ascore Correlates with Advanced Clinical Characteristics and Indicates Anoikis Resistance. (A) Differences in Ascore across BLCA clinical characteristics: age, stage, T stage, N stage, and M stage. (B) Volcano plot of DEGs between high and low Ascore groups, using the low Ascore group as control (| logFC | > 1, P < 0.05). (C) KEGG analysis comparing high and low Ascore groups. (D) Pearson correlation between Ascore and GSVA scores of specific anoikis-related gene sets. (*P < 0.05, **P< 0.01, ***P < 0.001, ****P < 0.0001).

** Additional file 6:** **Figure S6.** Distribution of Ascore and Comprising Genes in Single-Cell RNA Sequence Analysis. (A) Cell distribution of 8 patients before (left) and after (right) integration. (B) UMAP plot illustrating the distribution of seven main cell types in the integrated dataset, with doublets manually annotated. (C) Expression and distribution of marker genes in their corresponding cell types. (D) Dot plot displaying the average expression and percentage of four genes (CERCAM, EMP1, GNLY, PTPRR) and Ascore in different cell types.

** Additional file 7:** **Figure S7.** Immune Landscape Variations in BLCA Based on Ascore. (A) Comparison of immune cell proportions between Ascore groups in BLCA cohort via ESITIMATE. (B) Tumor mutational burden (TMB) differences between Ascore groups. (C, D) TIDE scores (C) and immunotherapy responders’ proportions (D) between Ascore groups. (*P < 0.05, **P < 0.01, ***P < 0.001, ****P < 0.0001).

** Additional file 8:** **Figure S8.** Ascore as an Independent Prognostic Indicator and Predicting Treatment Response in IMvigor210 Cohort. (A) Univariate and multivariate analysis showcasing Ascore's prognostic significance in bladder cancer patients in IMvigor210 cohort. (B) Survival outcomes within post-immunotherapy urothelial cancer patients relative to different Ascore groups. (C) Univariate and multivariate analysis showcasing Ascore's prognostic significance in urothelial cancer patients in IMvigor210 cohort. (D) ROC analysis of Ascore's predictive performance for survival against other prognostic factors like ECOG and liver metastasis in urothelial cancer. (E) Response rates to immunotherapy in urothelial cancer patients based on Ascore groups. (F) ROC analysis illustrating Ascore’s predictive accuracy for immunotherapy response in urothelial cancer. (*P< 0.05, **P < 0.01, ***P < 0.001; ECOG: Eastern Cooperative Oncology Group; ICIs: Immune Checkpoint Inhibitors; PD: progressive disease; SD: stable disease; PR: partial response; CR: complete response; TMB: Tumor Mutational Burden; IC: Immune Cell).

** Additional file 9:** **Figure S9.** Univariate and multivariate Cox Regression Analyses in Gulou-Cohort1. Univariate Cox regression analysis and multivariate Cox regression analysis showing the relationship between various clinical parameters (including lymph node metastasis, ECOG score, CTC count, and Ascore) and patient survival. 

** Additional file 10:** **Figure S10.** Immunohistochemical Scoring Criteria and PD-L1 Expression of Tumor-Infiltrating Immune Cells in Bladder Cancer Patients Receiving Anti-PD-1 Therapy. (A) IHC scoring criteria using H-score for CERCAM, EMP1, GNLY, and PTPRR. Intensity levels: “-” indicates no staining, “+” indicates weak staining, “++” indicates moderate staining, and “+++” indicates strong staining. (B) PD-L1 expression scoring criteria, highlighting the percentage of positively stained immune cells (right, indicated by black arrows). (C) Representative IHC images of PD-L1 in immune cells from patients 4 and 7. Patient 4, responsive to anti-PD-1 therapy, displayed PD-L1 expression at IC2 level, while patient 7, non-responsive, showed IC3 level. Black arrows pinpoint PD-L1-positive immune cells. (Scale bars = 100μm).

** Additional file 11:** **Figure S11.** Evaluation of previously established anoikis-related prognostic model in the IMvigor210 cohort. (A) Survival outcomes among patients in the immunotherapy cohort, categorized based on different signature scores derived from PMID: 37275895. (B) Response rates to immunotherapy in urothelial cancer patients based on signature groups.

** Additional file 12:** **Figure S12.** Immunohistochemical Control Validation for Ascore Assessment. (A) Hematoxylin and Eosin (H&E) staining of the amniotic coil. This panel provides an overview of the tissue morphology. (B-E) Immunohistochemical staining of the amniotic coil with each of the four antibodies used in our study. Each panel corresponds to a specific antibody, showcasing the distinct staining patterns and intensities.

** Additional file 13:** **Table S1.** 332 ARGs expression between normal and tumor bladder tissues.

** Additional file 14:** **Table S2.** Clinical characteristics and Ascore groups of patients in Gulou-Cohort1.

** Additional file 15:** **Table S3.** Immunotherapy response and Ascore groups of patients in Gulou-Cohort2.

** Additional file 16:** **Table S4.** Summary of studies exploring the link between anoikis and immunotherapy in cancer.

## Data Availability

The data that support the findings of this study are available from the corresponding author upon reasonable request.

## Abbreviations

BLCA: Bladder Cancer
NMIBC: Non-Muscle-Invasive Bladder Cancer
MIBC: Muscle-Invasive Bladder Cancer
RC: Radical Cystectomy
ICIs: Immune Checkpoint Inhibitors
ARGs: Anoikis-Related Genes
CTC: Circulating Tumor Cell
TIME: Tumor Immune Microenvironment
TMB: Tumor Mutation Burden
ECM: Extracellular Matrix
IC: Immune Cell
OS: Overall Survival
DSS: Disease-Free Survival
PFI: Progression-Free Interval
DFI: Disease-Free Interval
CR: Complete Response
PR: Partial Response
SD: Stable Disease
PD: Progression Disease
TCGA: The Cancer Genome Atlas
GEO: Gene Expression Omnibus
GO: Gene Ontology
KEGG: Kyoto Encyclopedia of Genes and Genomes
GSEA: Gene Set Enrichment Analysis
GSVA: Gene Set Variation Analysis
MSigDB: The Molecular Signatures Database
ssGSEA: Single Sample Gene Set Enrichment Analysis
TIDE: Tumor Immune Dysfunction and Exclusion
LASSO: Least Absolute Shrinkage and Selection Operator
NAT: Neoadjuvant Therapy
TURBT: Transurethral Resection of the Bladder Tumor
IHC: Immunohistochemistry
K-M: Kaplan-Meier
DEGs: Differentially Expressed Genes
