# Supplementary figures and images for: Multi-cohort validation of Ascore: an anoikis-based prognostic signature for predicting disease progression and immunotherapy response in bladder cancer
Source: Mol Cancer. 2024 Feb 10;23:30. doi: 10.1186/s12943-024-01945-9 (PMC10858533; doi:10.1186/s12943-024-01945-9)

Altered in 314 (81.56%) of 385 samples.

**A**

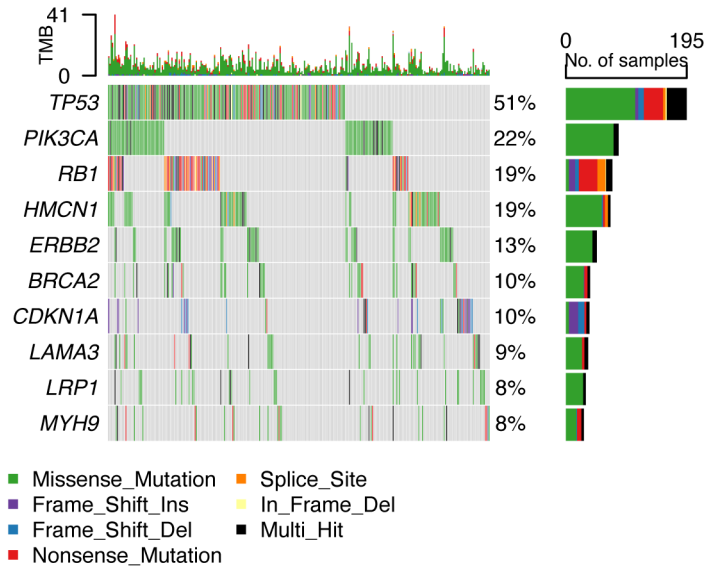

**B**

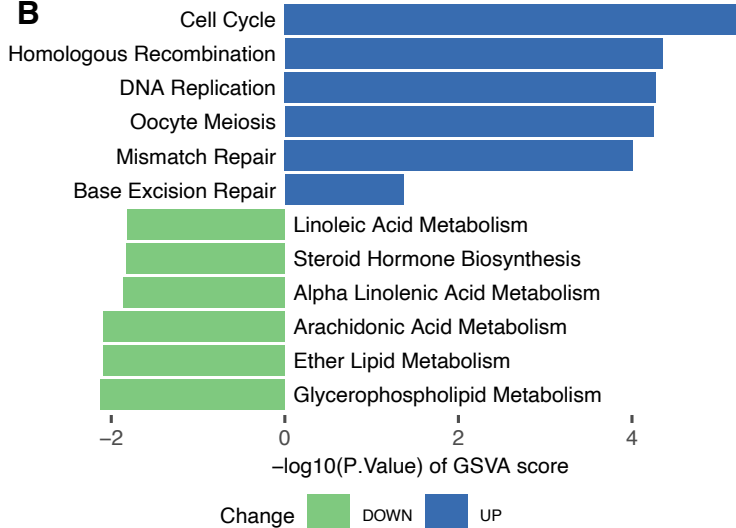

**D**

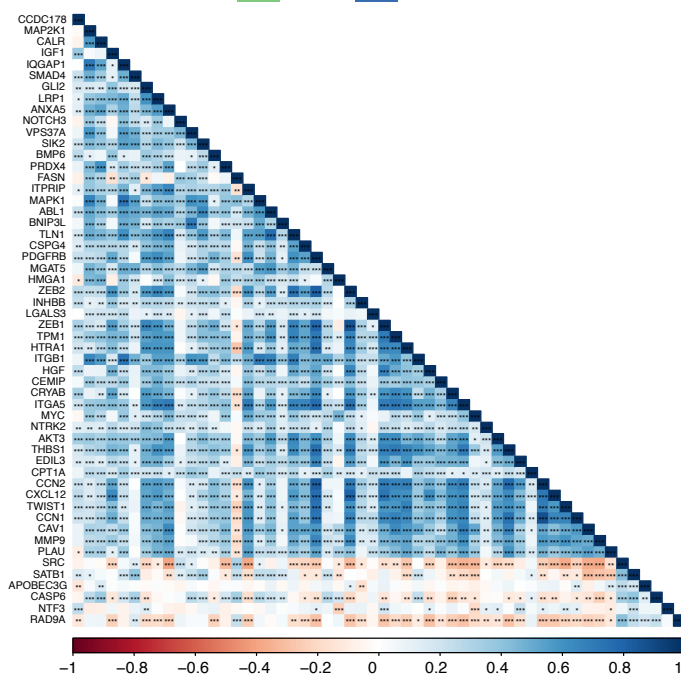

**C**

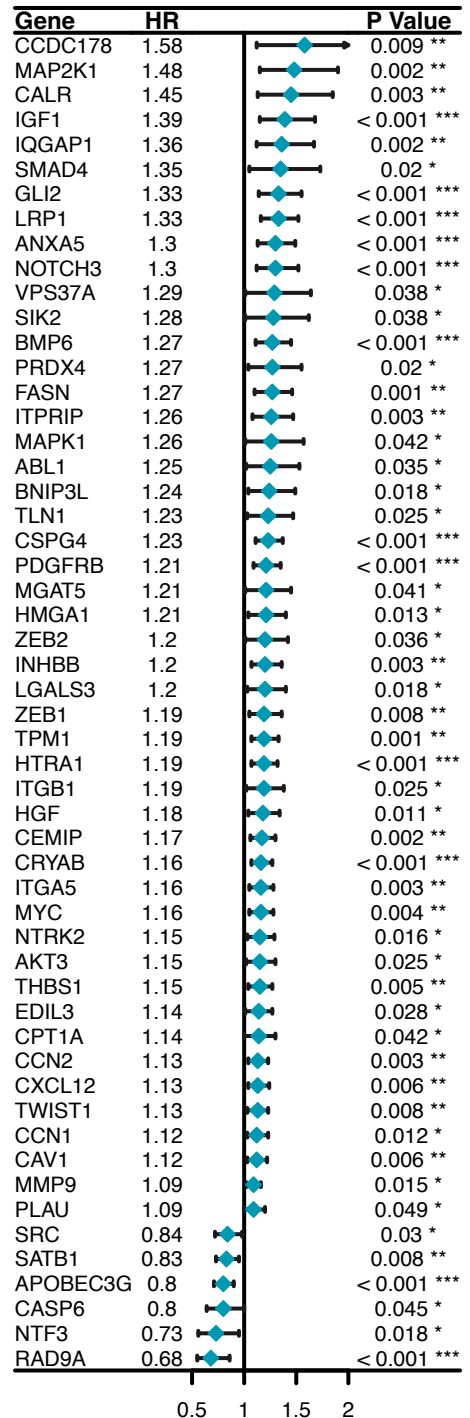

Supplement: Supplementary file 1 — Additional file 1: Figure S1. Mutation Patterns and Expression of Anoikis-related Genes (ARGs) in BLCA. (A) Top 10 mutated ARGs in the BLCA samples with mutations. (B) GSVA result showing differences in KEGG pathways between the Wild and Mutant groups. Pathways in blue/green indicate upregulation in the Mutant/Wild groups, respectively. (C) Forest plot of 54 prognostic ARGs. (D) Correlation plot illustrating interactions among the 54 prognosis-related ARGs. (*P < 0.05, **P < 0.01, ***P < 0.001). [file 12943_2024_1945_MOESM1_ESM.pdf]

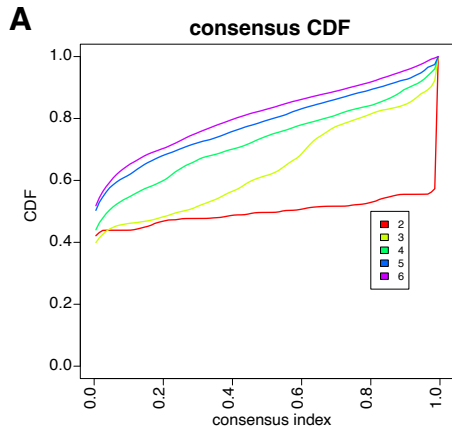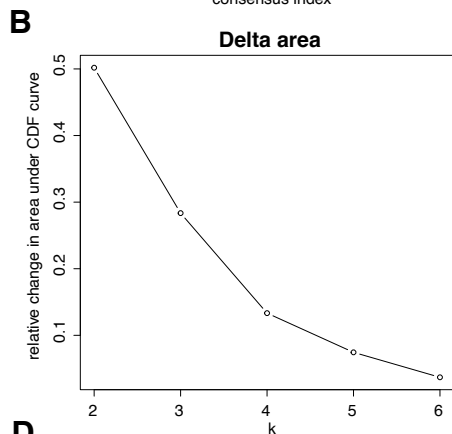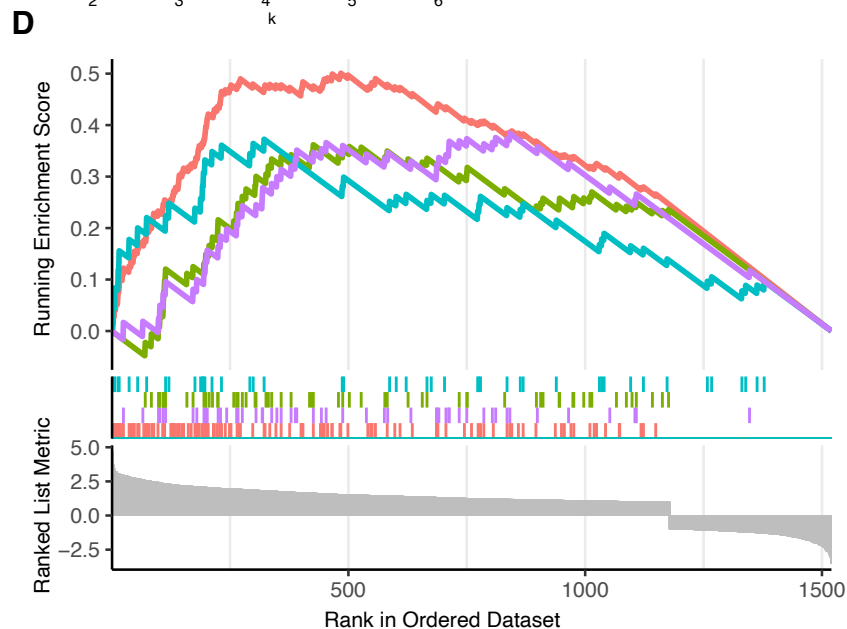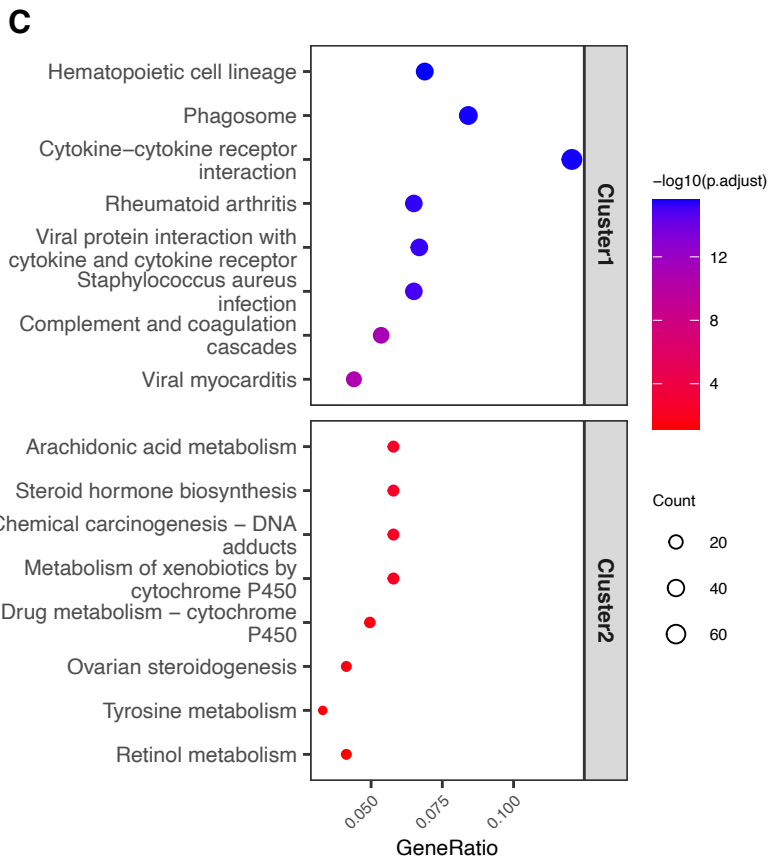

Supplement: Supplementary file 2 — Additional file 2: Figure S2. Consensus Clustering and inter-Cluster Differences. (A) Cumulative distribution function (CDF) plots illustrating consensus distribution with varying k values. (B) Delta area plot for relative change in the area under CDF curve. (C) KEGG analysis comparison between the two clusters. (D) GSEA results comparison, using Cluster 2 as the control group. [file 12943_2024_1945_MOESM2_ESM.pdf]

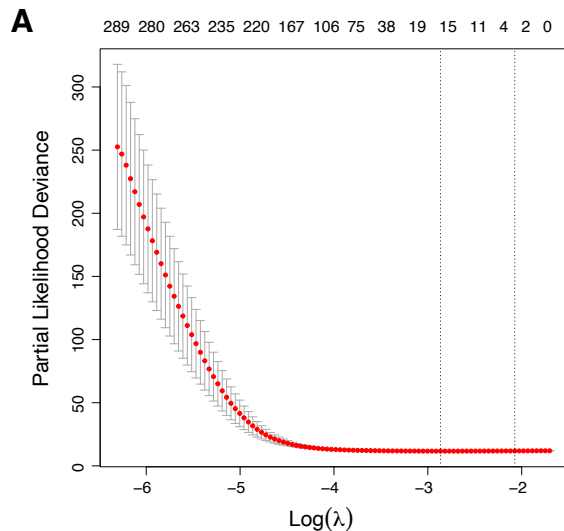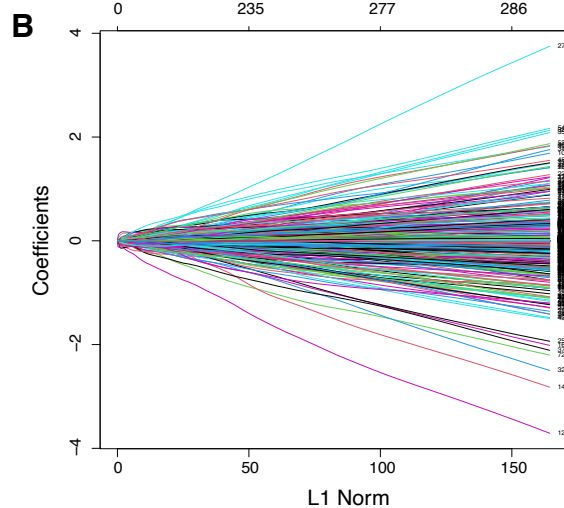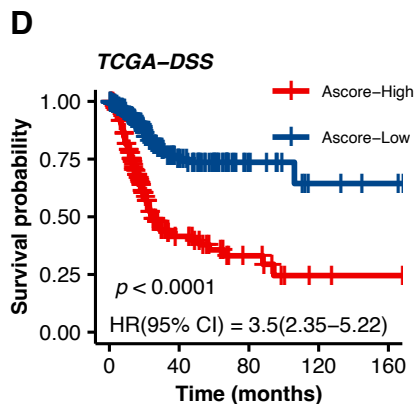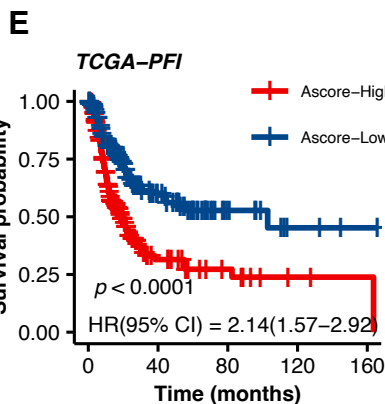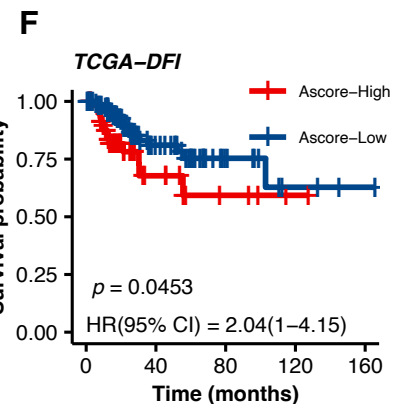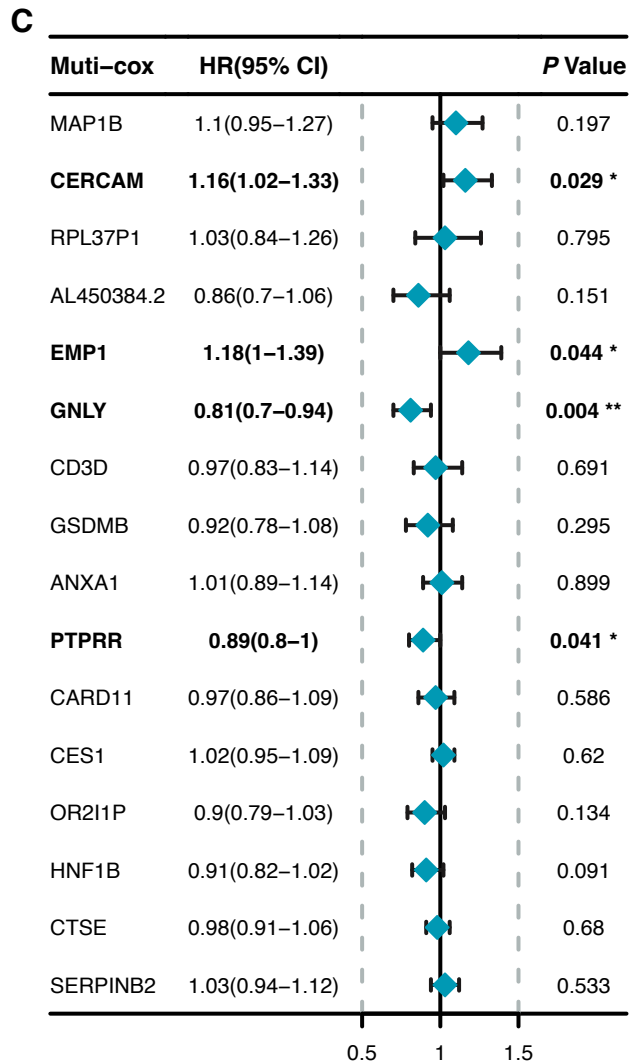

Supplement: Supplementary file 3 — Additional file 3: Figure S3. Construction of an ARGs-based Prognostic Signature. (A-B) LASSO regression with 10-fold cross-validation identified 16 prognostic ARGs following univariate Cox regression analysis. (C) Multivariate Cox regression analysis of the 16 genes shortlisted by LASSO regression. (D-F) Kaplan-Meier analysis comparing Disease-Free Survival (DSS) (D), Progression-Free Interval (PFI) (E), and Disease-Free Interval (DFI) (F) between high and low Ascore groups in BLCA. (*P < 0.05, **P < 0.01). [file 12943_2024_1945_MOESM3_ESM.pdf]

A

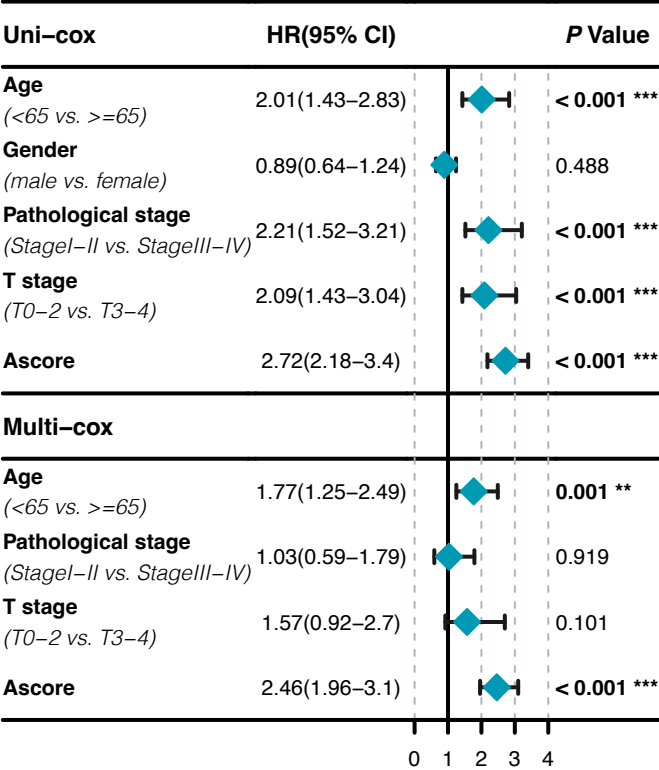

B

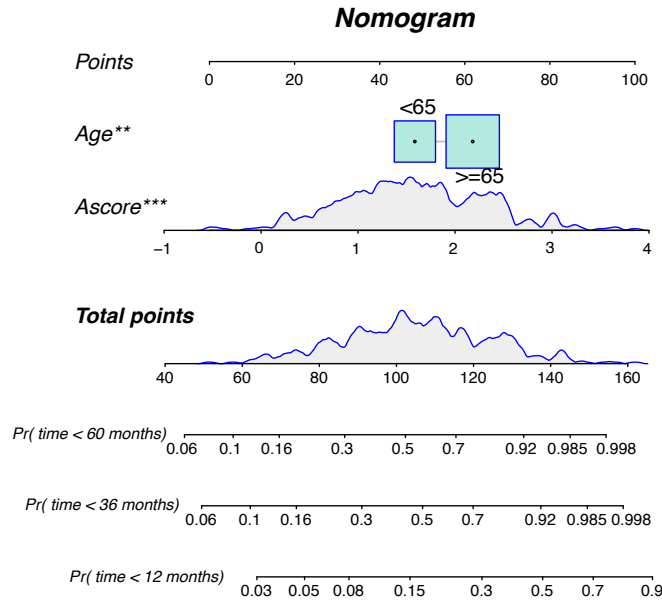

C

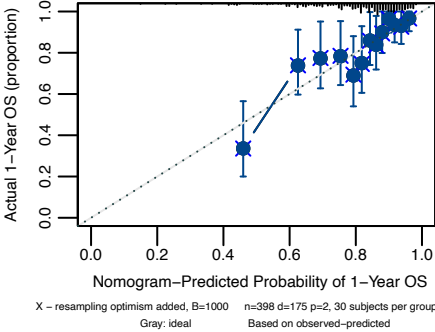

D

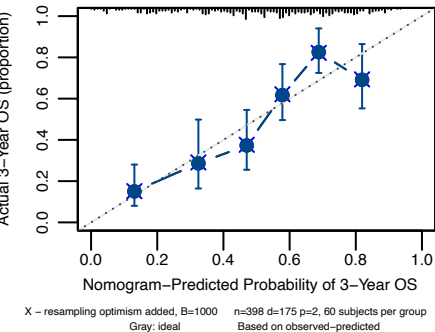

E

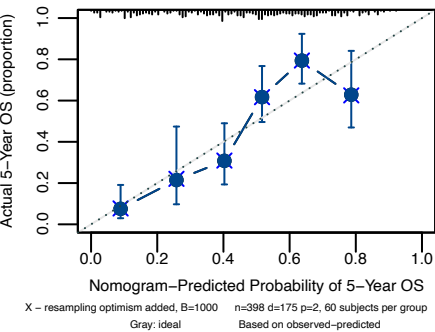

F

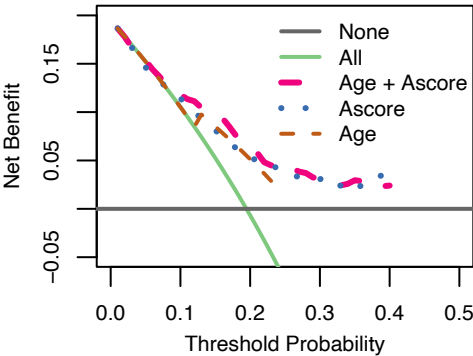

Supplement: Supplementary file 4 — Additional file 4: Figure S4. BLCA Survival Prediction Nomogram Based on Ascore. (A) Univariate Cox regression analysis of clinical characteristics and Ascore. Factors with P < 0.05 were included in subsequent multivariate Cox regression analysis. (B) Nomogram incorporating age and Ascore, utilized for 1, 3, and 5-year survival predictions. (C-E) Calibration curves at 1, 3, and 5 years, respectively, demonstrating nomogram's predictive accuracy. (F) Decision curve analysis (DCA) evaluating the clinical utility of the nomogram. (**P< 0.01, ***P < 0.001). [file 12943_2024_1945_MOESM4_ESM.pdf]

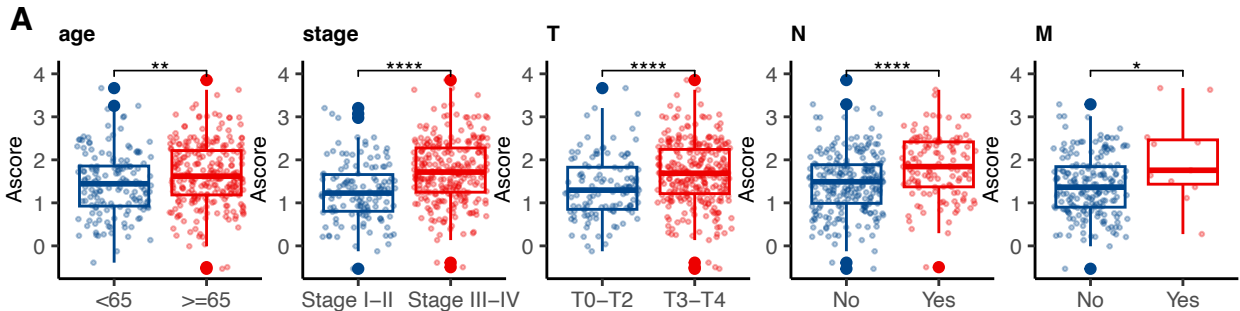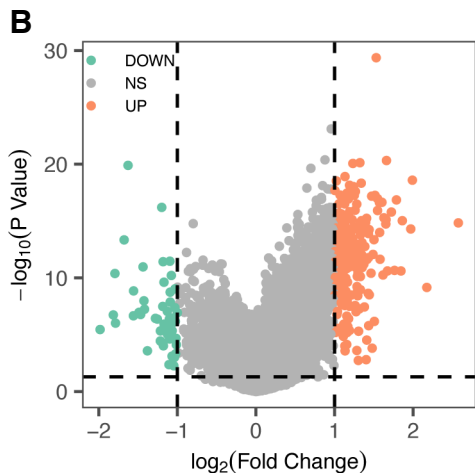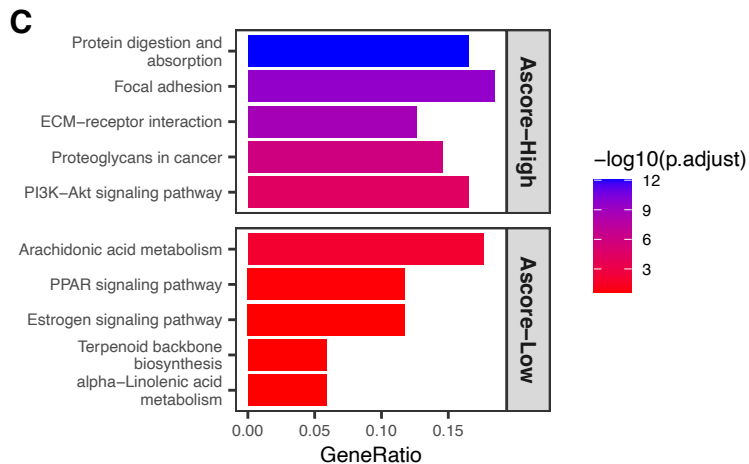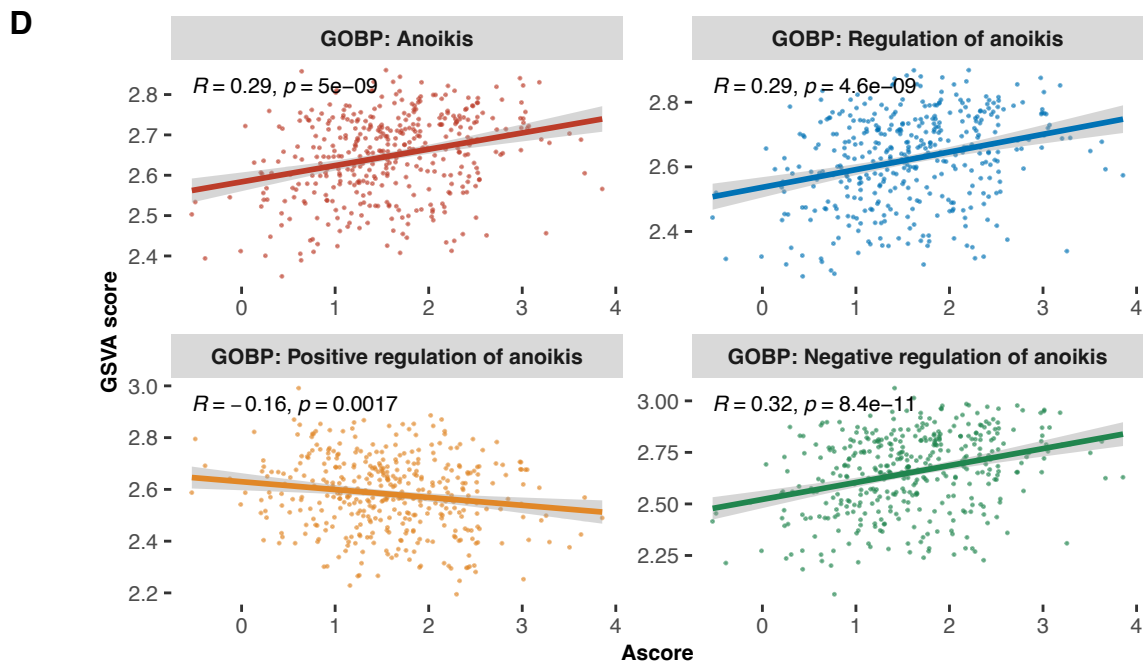

Supplement: Supplementary file 5 — Additional file 5: Figure S5. High Ascore Correlates with Advanced Clinical Characteristics and Indicates Anoikis Resistance. (A) Differences in Ascore across BLCA clinical characteristics: age, stage, T stage, N stage, and M stage. (B) Volcano plot of DEGs between high and low Ascore groups, using the low Ascore group as control (| logFC | > 1, P < 0.05). (C) KEGG analysis comparing high and low Ascore groups. (D) Pearson correlation between Ascore and GSVA scores of specific anoikis-related gene sets. (*P < 0.05, **P< 0.01, ***P < 0.001, ****P < 0.0001). [file 12943_2024_1945_MOESM5_ESM.pdf]

**A**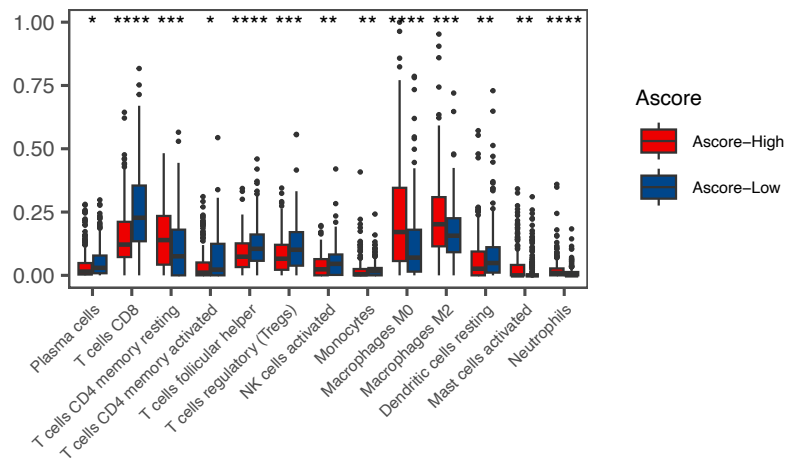**B**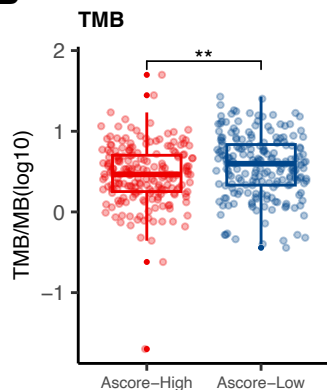**C**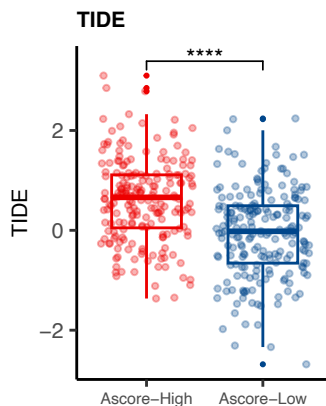**D**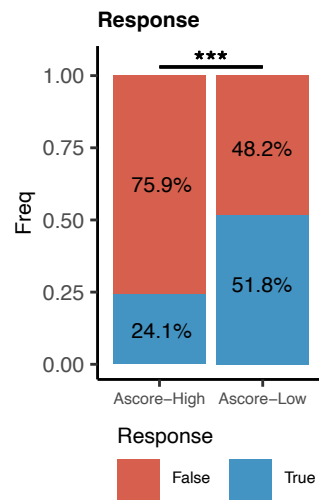

Supplement: Supplementary file 7 — Additional file 7: Figure S7. Immune Landscape Variations in BLCA Based on Ascore. (A) Comparison of immune cell proportions between Ascore groups in BLCA cohort via ESITIMATE. (B) Tumor mutational burden (TMB) differences between Ascore groups. (C, D) TIDE scores (C) and immunotherapy responders’ proportions (D) between Ascore groups. (*P < 0.05, **P < 0.01, ***P < 0.001, ****P < 0.0001). [file 12943_2024_1945_MOESM7_ESM.pdf]

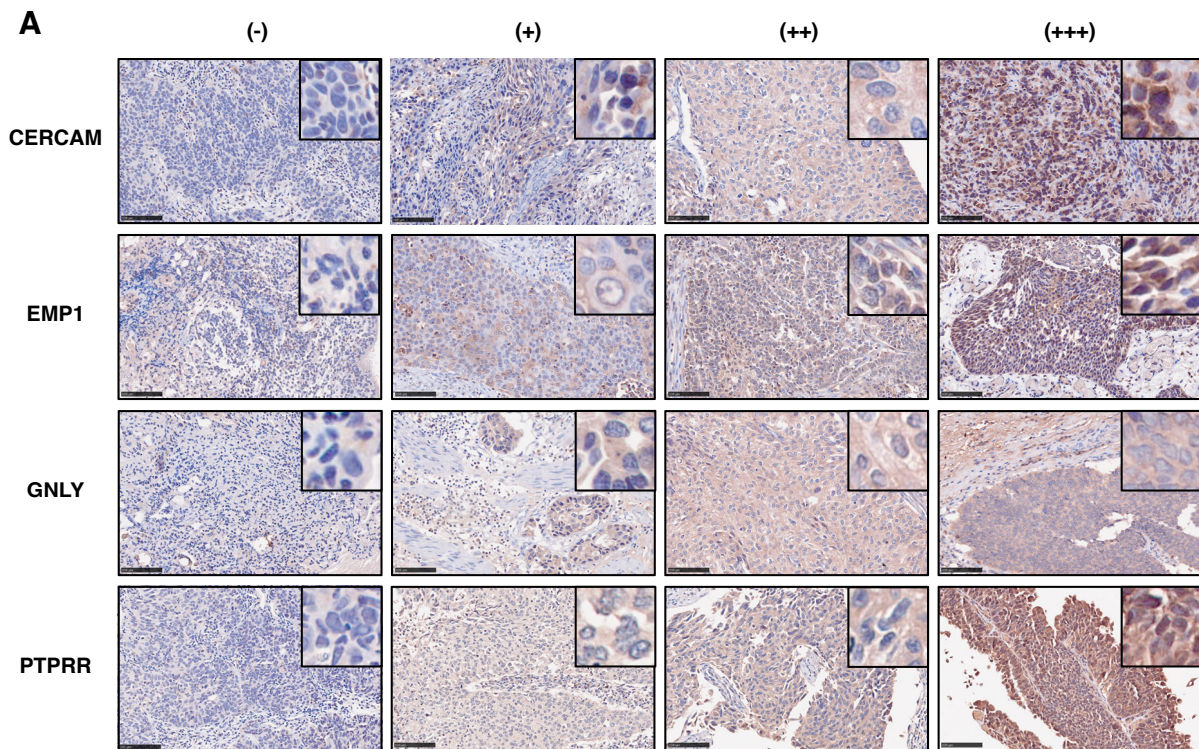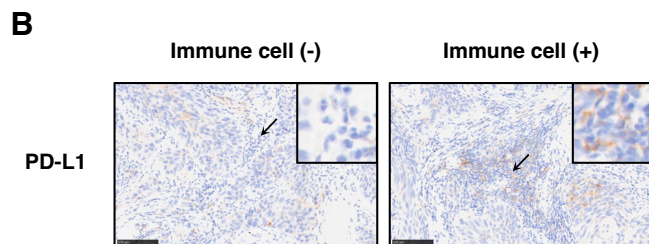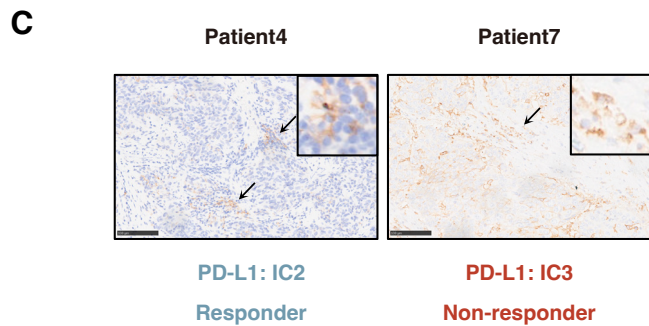

Supplement: Supplementary file 10 — Additional file 10: Figure S10. Immunohistochemical Scoring Criteria and PD-L1 Expression of Tumor-Infiltrating Immune Cells in Bladder Cancer Patients Receiving Anti-PD-1 Therapy. (A) IHC scoring criteria using H-score for CERCAM, EMP1, GNLY, and PTPRR. Intensity levels: “-” indicates no staining, “+” indicates weak staining, “++” indicates moderate staining, and “+++” indicates strong staining. (B) PD-L1 expression scoring criteria, highlighting the percentage of positively stained immune cells (right, indicated by black arrows). (C) Representative IHC images of PD-L1 in immune cells from patients 4 and 7. Patient 4, responsive to anti-PD-1 therapy, displayed PD-L1 expression at IC2 level, while patient 7, non-responsive, showed IC3 level. Black arrows pinpoint PD-L1-positive immune cells. (Scale bars = 100μm). [file 12943_2024_1945_MOESM10_ESM.pdf]

A

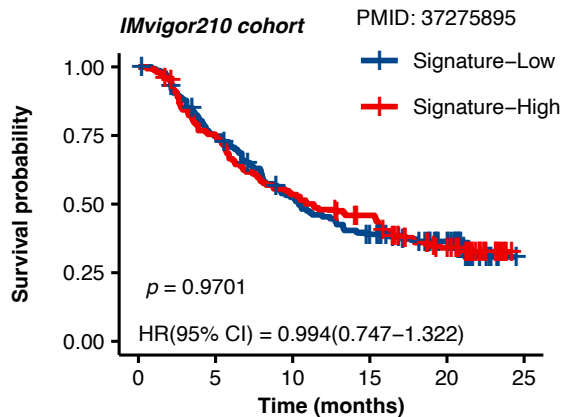

**Number at risk**

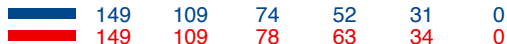

B

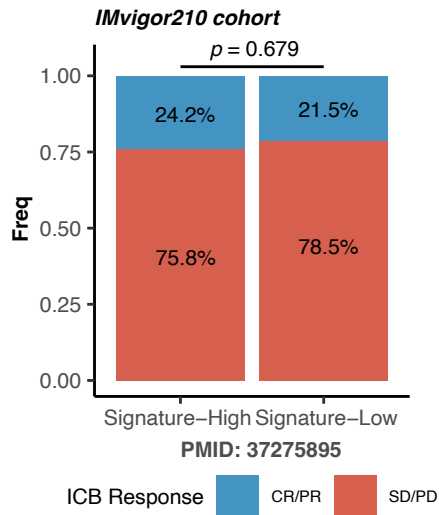

Supplement: Supplementary file 11 — Additional file 11: Figure S11. Evaluation of previously established anoikis-related prognostic model in the IMvigor210 cohort. (A) Survival outcomes among patients in the immunotherapy cohort, categorized based on different signature scores derived from PMID: 37275895. (B) Response rates to immunotherapy in urothelial cancer patients based on signature groups. [file 12943_2024_1945_MOESM11_ESM.pdf]

**A**

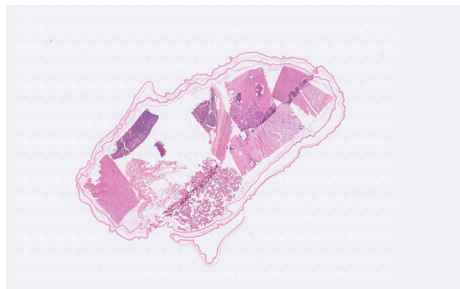

H&E

**B**

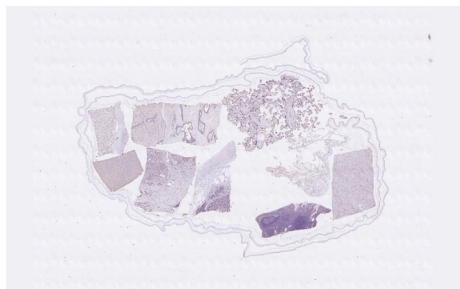

CERCAM

**C**

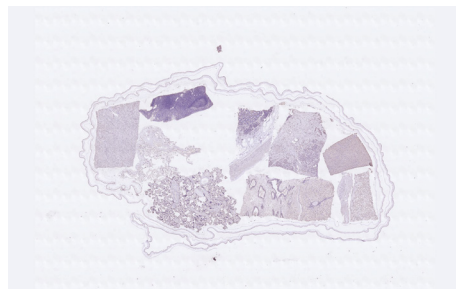

EMP1

**D**

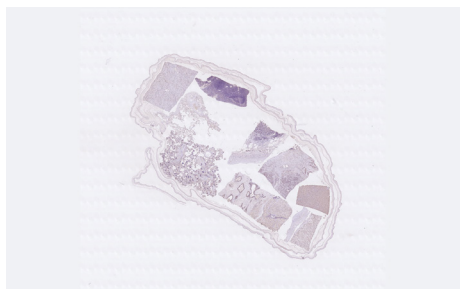

GNLY

**E**

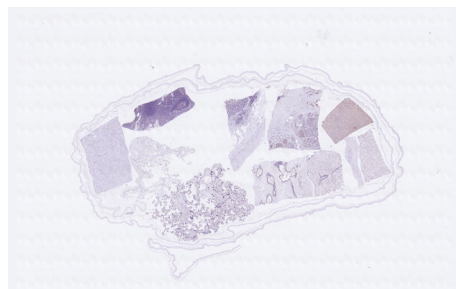

PTPRR

Supplement: Supplementary file 12 — Additional file 12: Figure S12. Immunohistochemical Control Validation for Ascore Assessment. (A) Hematoxylin and Eosin (H&E) staining of the amniotic coil. This panel provides an overview of the tissue morphology. (B-E) Immunohistochemical staining of the amniotic coil with each of the four antibodies used in our study. Each panel corresponds to a specific antibody, showcasing the distinct staining patterns and intensities. [file 12943_2024_1945_MOESM12_ESM.pdf]
