# Supplementary material for: Multi-cohort validation of Ascore: an anoikis-based prognostic signature for predicting disease progression and immunotherapy response in bladder cancer
Source: Mol Cancer. 2024 Feb 10;23:30. doi: 10.1186/s12943-024-01945-9 (PMC10858533; doi:10.1186/s12943-024-01945-9)
Supplement: Supplementary file 6 — Additional file 6: Figure S6. Distribution of Ascore and Comprising Genes in Single-Cell RNA Sequence Analysis. (A) Cell distribution of 8 patients before (left) and after (right) integration. (B) UMAP plot illustrating the distribution of seven main cell types in the integrated dataset, with doublets manually annotated. (C) Expression and distribution of marker genes in their corresponding cell types. (D) Dot plot displaying the average expression and percentage of four genes (CERCAM, EMP1, GNLY, PTPRR) and Ascore in different cell types. [file 12943_2024_1945_MOESM6_ESM.pdf]

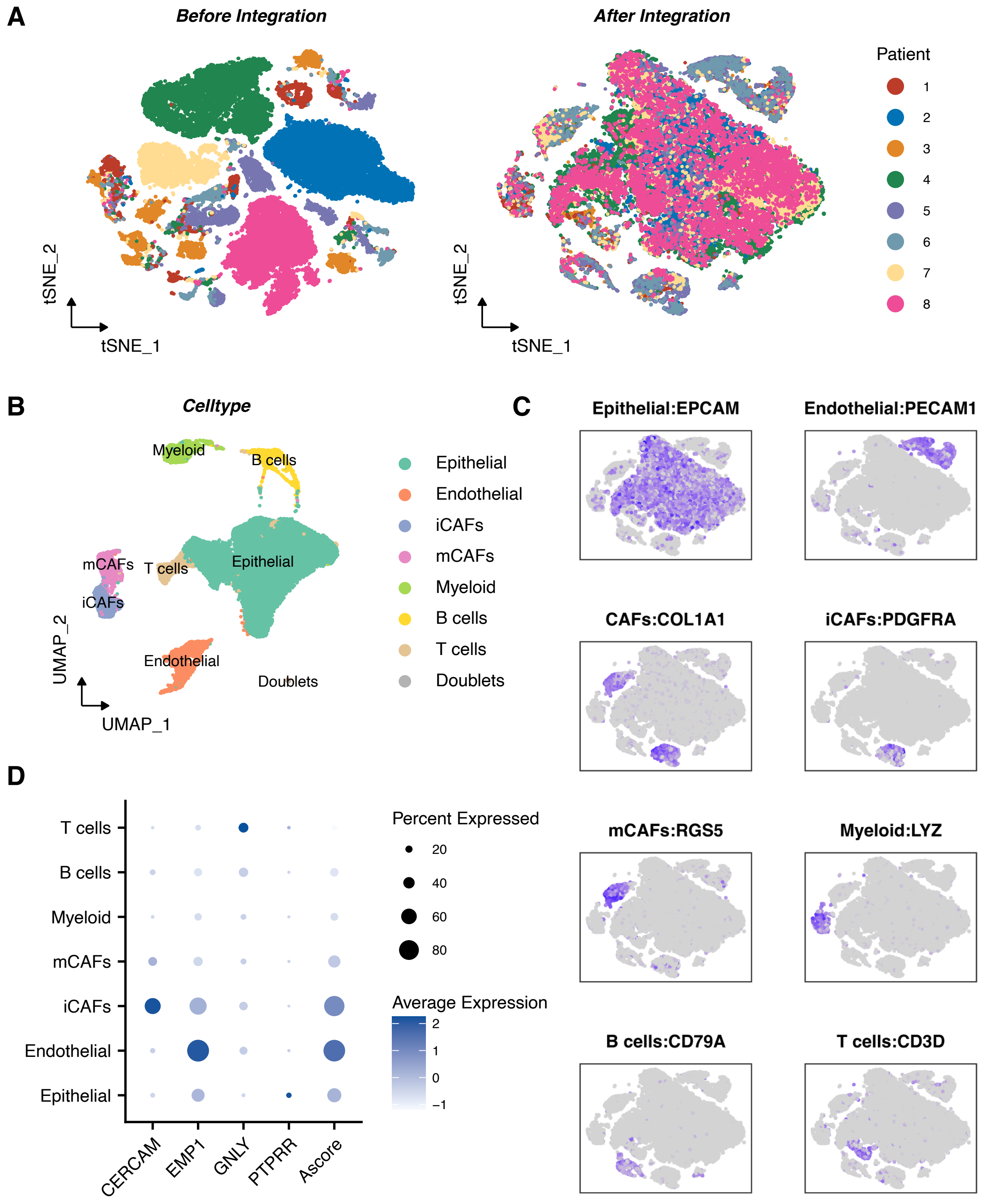
A
Before Integration
After Integration
Patient
1
2
3
4
5
6
7
8
tSNE_2
tSNE_1
B
Celltype
Myeloid
B cells
mCAFs
T cells
Epithelial
iCAFs
Endothelial
Doublets
UMAP_2
UMAP_1
Epithelial
Endothelial
iCAFs
mCAFs
Myeloid
B cells
T cells
Doublets
C
Epithelial:EPCAM
Endothelial:PECAM1
CAFs:COL1A1
iCAFs:PDGFRA
mCAFs:RGS5
Myeloid:LYZ
B cells:CD79A
T cells:CD3D
D
T cells
B cells
Myeloid
mCAFs
iCAFs
Endothelial
Epithelial
CERCAM
EMP1
GNLY
PTPRR
Ascore
Percent Expressed
20
40
60
80
Average Expression
2
1
0
−1
