# Supplementary material for: Multi-cohort validation of Ascore: an anoikis-based prognostic signature for predicting disease progression and immunotherapy response in bladder cancer
Source: Mol Cancer. 2024 Feb 10;23:30. doi: 10.1186/s12943-024-01945-9 (PMC10858533; doi:10.1186/s12943-024-01945-9)
Supplement: Supplementary file 8 — Additional file 8: Figure S8. Ascore as an Independent Prognostic Indicator and Predicting Treatment Response in IMvigor210 Cohort. (A) Univariate and multivariate analysis showcasing Ascore's prognostic significance in bladder cancer patients in IMvigor210 cohort. (B) Survival outcomes within post-immunotherapy urothelial cancer patients relative to different Ascore groups. (C) Univariate and multivariate analysis showcasing Ascore's prognostic significance in urothelial cancer patients in IMvigor210 cohort. (D) ROC analysis of Ascore's predictive performance for survival against other prognostic factors like ECOG and liver metastasis in urothelial cancer. (E) Response rates to immunotherapy in urothelial cancer patients based on Ascore groups. (F) ROC analysis illustrating Ascore’s predictive accuracy for immunotherapy response in urothelial cancer. (*P< 0.05, **P < 0.01, ***P < 0.001; ECOG: Eastern Cooperative Oncology Group; ICIs: Immune Checkpoint Inhibitors; PD: progressive disease; SD: stable disease; PR: partial response; CR: complete response; TMB: Tumor Mutational Burden; IC: Immune Cell). [file 12943_2024_1945_MOESM8_ESM.pdf]

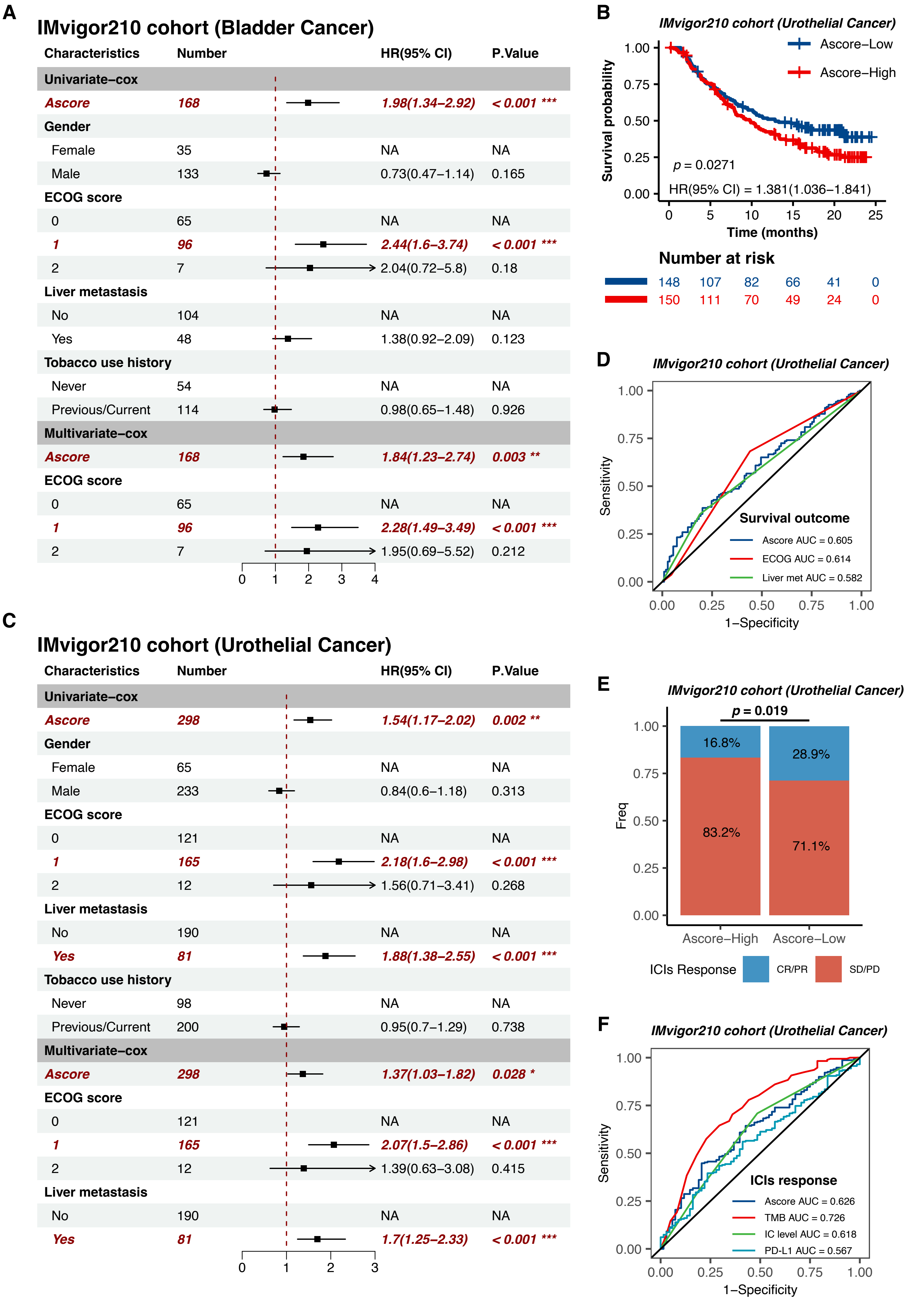
A
IMvigor210 cohort (Bladder Cancer)
Characteristics | Number | HR(95% CI) | P.Value
Univariate−cox
Ascore | 168 | 1.98(1.34−2.92) | < 0.001 ***
Gender
Female | 35 | NA | NA
Male | 133 | 0.73(0.47−1.14) | 0.165
ECOG score
0 | 65 | NA | NA
1 | 96 | 2.44(1.6−3.74) | < 0.001 ***
2 | 7 | 2.04(0.72−5.8) | 0.18
Liver metastasis
No | 104 | NA | NA
Yes | 48 | 1.38(0.92−2.09) | 0.123
Tobacco use history
Never | 54 | NA | NA
Previous/Current | 114 | 0.98(0.65−1.48) | 0.926
Multivariate−cox
Ascore | 168 | 1.84(1.23−2.74) | 0.003 **
ECOG score
0 | 65 | NA | NA
1 | 96 | 2.28(1.49−3.49) | < 0.001 ***
2 | 7 | 1.95(0.69−5.52) | 0.212
0 1 2 3 4
B
IMvigor210 cohort (Urothelial Cancer)
Ascore−Low
Ascore−High
Survival probability
1.00 0.75 0.50 0.25 0.00
p = 0.0271
HR(95% CI) = 1.381(1.036−1.841)
0 5 10 15 20 25
Time (months)
Number at risk
148 107 82 66 41 0
150 111 70 49 24 0
C
IMvigor210 cohort (Urothelial Cancer)
Characteristics | Number | HR(95% CI) | P.Value
Univariate−cox
Ascore | 298 | 1.54(1.17−2.02) | 0.002 **
Gender
Female | 65 | NA | NA
Male | 233 | 0.84(0.6−1.18) | 0.313
ECOG score
0 | 121 | NA | NA
1 | 165 | 2.18(1.6−2.98) | < 0.001 ***
2 | 12 | 1.56(0.71−3.41) | 0.268
Liver metastasis
No | 190 | NA | NA
Yes | 81 | 1.88(1.38−2.55) | < 0.001 ***
Tobacco use history
Never | 98 | NA | NA
Previous/Current | 200 | 0.95(0.7−1.29) | 0.738
Multivariate−cox
Ascore | 298 | 1.37(1.03−1.82) | 0.028 *
ECOG score
0 | 121 | NA | NA
1 | 165 | 2.07(1.5−2.86) | < 0.001 ***
2 | 12 | 1.39(0.63−3.08) | 0.415
Liver metastasis
No | 190 | NA | NA
Yes | 81 | 1.7(1.25−2.33) | < 0.001 ***
0 1 2 3
D
IMvigor210 cohort (Urothelial Cancer)
Sensitivity
1−Specificity
Survival outcome
Ascore AUC = 0.605
ECOG AUC = 0.614
Liver met AUC = 0.582
E
IMvigor210 cohort (Urothelial Cancer)
p = 0.019
Freq
16.8%
83.2%
28.9%
71.1%
Ascore−High
Ascore−Low
ICIs Response
CR/PR
SD/PD
F
IMvigor210 cohort (Urothelial Cancer)
Sensitivity
1−Specificity
ICIs response
Ascore AUC = 0.626
TMB AUC = 0.726
IC level AUC = 0.618
PD-L1 AUC = 0.567
