# Supplementary material for: Multi-cohort validation of Ascore: an anoikis-based prognostic signature for predicting disease progression and immunotherapy response in bladder cancer
Source: Mol Cancer. 2024 Feb 10;23:30. doi: 10.1186/s12943-024-01945-9 (PMC10858533; doi:10.1186/s12943-024-01945-9)

Gulou–Cohort1

Characteristics	Number	HR(95% CI)	*P*.Value
**Univariate–cox**			
Age	62	1.03(0.94–1.12)	0.557
Gender			
Female	25	NA	NA
Male	37	0.51(0.14–1.89)	0.31
Stage			
II	34	NA	NA
III	28	7.75(0.96–62.33)	0.054
T_stage			
T2	37	NA	NA
T3	25	2.53(0.63–10.17)	0.191
N_stage			
N0	51	NA	NA
N1	***11***	***12.43(2.56–60.27)***	***0.002 *****
ECOG score			
0	46	NA	NA
1	***16***	***6.65(1.75–25.28)***	***0.005 *****
Ascore	***62***	***3.76(1.69–8.39)***	***0.001 *****
CTC counts	***62***	***1.09(1.01–1.16)***	***0.018 ****
**Multivariate–cox**			
N_stage			
N0	51	NA	NA
N1	11	2.88(0.38–21.55)	0.303
ECOG score			
0	46	NA	NA
1	***16***	***6.46(1.3–32.07)***	***0.022 ****
Ascore	***62***	***2.89(1.09–7.72)***	***0.034 ****
CTC counts	62	1.03(0.92–1.14)	0.638

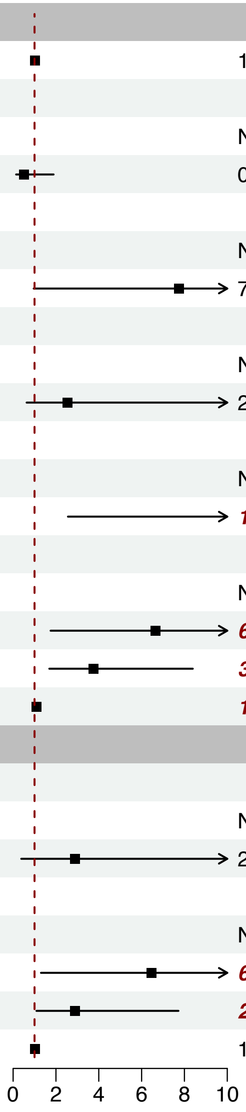

Supplement: Supplementary file 9 — Additional file 9: Figure S9. Univariate and multivariate Cox Regression Analyses in Gulou-Cohort1. Univariate Cox regression analysis and multivariate Cox regression analysis showing the relationship between various clinical parameters (including lymph node metastasis, ECOG score, CTC count, and Ascore) and patient survival. [file 12943_2024_1945_MOESM9_ESM.pdf]
